# Engineering Edible Double Network Hydrogels with Abalone- and Squid-like Textures from Carrageenan and Konjac Glucomannan

**DOI:** 10.3390/foods14173140

**Published:** 2025-09-08

**Authors:** Jingwen Zhao, Mengjia Du, Yiguo Zhao, Yapeng Fang

**Affiliations:** 1 Department of Food Science and Engineering, School of Agriculture and Biology, Shanghai Jiao Tong University, Shanghai 200240, China; jwzhao623@sjtu.edu.cn (J.Z.); dumengjia16@163.com (M.D.); 2 School of Health Science and Engineering, University of Shanghai for Science and Technology, Shanghai 200093, China

**Keywords:** carrageenan, konjac glucomannan, double network hydrogels, mechanical performance, biomimetic seafood

## Abstract

The development of edible hydrogels with both high strength and toughness remains a considerable challenge. Herein, we report an innovative and straightforward method to prepare robust kappa-carrageenan/konjac glucomannan (κ-car/KGM) double network hydrogels (DNs) through a single heating-cooling cycle followed by immersion in an Na_2_CO_3_ solution. This method effectively tuned the crosslinking densities of both the rigid κ-car-k^+^ first network and the ductile KGM second network. The resulting κ-car-k^+^/KGM-1:2 DNs demonstrated outstanding mechanical properties, exhibiting compressive strength approximately 10- and 20-fold greater than that of the corresponding single network (SN) κ-car-k^+^ and KGM hydrogels, respectively, accompanied by remarkable toughness. This enhancement is attributed to a sequential failure mechanism where the rigid κ-car-k^+^ network fractures first to dissipate energy, while the ductile KGM network remains intact to maintain structural integrity. Furthermore, the sensory profile of the κ-car-k^+^/KGM-1:2 DNs was remarkably similar to that of squid across all five evaluated attributes (hardness, elasticity, chewiness, brittleness, and palatability). Similarly, the κ-car-k^+^/KGM-1:1 DNs closely matched the sensory profile of abalone, particularly in elasticity, chewiness, brittleness, and palatability. These findings suggest the prepared edible DNs have great potential to simulate high-chewing seafood, offering a new pathway for designing advanced food materials from natural polysaccharides.

## 1. Introduction

Hydrogels, which belong to the category of soft and moist materials, are three-dimensional polymeric structures that can hold large quantities of water [1,2]. However, hydrogels derived from natural polymers often exhibit inherently limited mechanical properties, which significantly constrain their practical applications, especially in demanding fields such as high-chewiness food analogues and biomedical materials requiring superior mechanical performance [3]. To overcome this limitation, the double network (DN) architecture has emerged as an extremely effective strategy for preparing hydrogels with exceptional strength and toughness [4]. The classic DN design comprises a densely cross-linked, brittle first network and a loosely cross-linked, ductile second network. Under deformation, the brittle network fractures to dissipate energy, while the ductile network maintains the overall structural integrity [5]. Although many high-performance DN hydrogels (DNs) have been developed using synthetic polymers, their limited biocompatibility and non-degradability pose significant barriers for edible or biomedical applications [6,7].

Therefore, research has shifted towards the preparation of DNs from food macromolecules such as polysaccharides and proteins [8,9,10,11]. However, the development of these natural DNs usually involves complex multi-step procedures, chemical modifications, or the production of hydrogels that are strong but lack toughness [12,13]. Building on this, our group previously fabricated several fully physical DNs and found that a neutral polysaccharide, konjac glucomannan (KGM), served as a particularly effective ductile network for improving both strength and toughness [5,13,14]. Moreover, it is well-established that a rigid polyelectrolyte network can be a more efficient energy dissipator due to its higher swelling ratio and chain rigidity [15]. Therefore, we hypothesized that pairing the highly ductile neutral KGM network with a rigid polyelectrolyte first network, such as kappa carrageenan (κ-car), would form a synergistic system for achieving outstanding mechanical performance. In addition, both κ-car and KGM are annually renewable polysaccharides that require minimal land and water inputs and are fully biodegradable, providing a sustainable alternative to animal-derived gelling agents [16,17,18,19]. Their assembly into biomimetic architectures can reproduce the fibrous, elastic, and fracture behaviors of high-value seafood without invoking animal protein, thus addressing ethical and environmental concerns.

In this study, we report a facile and innovative sequential physical crosslinking strategy to fabricate robust κ-car-k^+^/KGM DNs. This method involved a simple heating-cooling cycle to form the κ-car-k^+^ network, followed by an alkaline immersion step to induce gelation of the KGM network. The mechanical properties, including compressive/tensile behavior and self-recovery capabilities, of the DNs were investigated, and then their underlying strengthening and toughening mechanisms were elucidated. Moreover, the potential of these edible DNs as high-chewiness seafood (squid and abalone) analogues was explored. To achieve this, the mechanical, textural, and sensory characteristics of the optimized hydrogels were comprehensively evaluated and benchmarked against natural abalone and squid samples using advanced statistical analyses. Squid and abalone were selected as target textures because they, respectively, represent dense, aligned muscle bundles (squid) and tough, nacre-like lamellar structures (abalone), both delivering pronounced chewiness that is challenging to replicate [20,21,22]. This work not only presents a novel material with outstanding mechanical properties but also provides a new paradigm for designing biomimetic food structures from sustainable polysaccharides.

## 2. Materials and Methods

### 2.1. Materials

Kappa carrageenan and konjac glucomannan were supplied by Beilian Biological Co., Ltd. (Shanghai, China). The potassium chloride (KCl) granules were provided by Sinopharm Chemical Reagent Co., Ltd. (Shanghai, China). The fresh squid and abalone were acquired from Oushang Supermarket in Shanghai. The sodium carbonate (Na_2_CO_3_) granules were purchased from Sinopharm Group Co., Ltd. (Shanghai, China).

### 2.2. Fabrication of κ-car-k^+^/KGM Double Networks and Thermally Processed Seafood Samples

The κ-car-k^+^/KGM DNs were synthesized following the procedure described by Chen et al. [23] with certain adjustments. The κ-car and KGM powders were uniformly dispersed in ultrapure water using magnetic stirring at 25 °C. The resulting mixture was then heated to 90 °C and maintained at this temperature for 20 min to ensure full dissolution. Following this, KCl granules were introduced into the mixture at 90 °C. The resulting solution was rapidly poured into cylindrical molds (10 mm in height and 14 mm in diameter) and cooled at 4 °C for 12 h to allow hydrogel formation. Subsequently, these hydrogels were completely immersed into Na_2_CO_3_ solution at 30 °C to obtain κ-car-k^+^/KGM DNs. The single network (SN) hydrogels based on κ-car-k^+^ or KGM were synthesized by following the same steps described for the double network (DN) hydrogels, with the exception that either KGM or κ-car powder was omitted. After synthesis, the resulting hydrogels were rinsed thoroughly with deionized water to eliminate any unreacted Na_2_CO_3_. The overall macromolecular solid content within the hydrogels was maintained at 2 wt%. The prepared DN hydrogels were labeled as κ-car-k^+^/KGM-x:y double networks, with the x:y ratio indicating the relative weight proportions of κ-car to KGM used.

Seafood samples (squid and abalone) were subjected to heating in ultrapure water at 99 °C for durations of 10, 20, and 30 min [24]. Subsequently, the samples were shaped into cylindrical forms measuring 3 mm in height and 14 mm in diameter and then placed in a refrigerator set at 4 °C for subsequent analysis.

### 2.3. Mechanical Properties Test

A texture analyzer (TA-XT. Plus, Stable Micro Systems, Godalming, Surrey, UK) equipped with a 50 kg load cell was used to measure the mechanical properties of the gels [23]. For the compression tests, a cylindrical hydrogel with a 14 mm diameter and 10 mm height was set on the lower plate and compressed by the upper plate at a strain rate of 0.25 mm/s. For the tensile tests, the samples were made into dumbbell shapes (total length and width were 18 and 8 mm, respectively; the length, width, and thickness of the narrow neck were 10, 6, and 2 mm, respectively) and stretched using a clamp attachment at a strain rate of 0.25 mm/s. For the loading–unloading tests, the strain rate was fixed at 0.25 mm/s. The trigger force was 3 g.

### 2.4. Texture Profile Test

Texture profile analysis (TPA) was conducted using an established method [25]. A texture analyzer (TA-XT Plus, Stable Micro Systems, Godalming, Surrey, UK), fitted with a 50 kg load cell and a P/36R cylindrical aluminum probe, was employed to evaluate the textural properties of the gel samples. The hydrogels were sectioned into cylindrical specimens with a diameter of 14 mm and a height of 3 mm. These samples were then compressed to 30% of their original height, with pre-test, test, and post-test speeds all set at 1 mm/s, to determine TPA parameters, including hardness, springiness, and chewiness. To minimize surface friction and adhesion during testing, paraffin oil was applied as a lubricant. A 2-s interval was maintained between the two compression cycles. Additionally, the puncture force was measured using a P/2 cylindrical aluminum probe, with a puncture depth of 5 mm and a trigger force of 3.0 g.

### 2.5. Water Retention Ability and Swelling Characteristics Test

Water retention capacity of hydrogel and seafood specimens was detected according to a previous method [26]. Hydrogel and seafood specimens were subjected to centrifugation at 5000× *g* for 20 min at 25 °C. Following centrifugation, the supernatant was carefully decanted, and the mass of the remaining sample was recorded. The water retention capacity was then determined using the following equation:

$$\mathrm{Water}\;\mathrm{retention}\;\mathrm{capacity}\;=\frac{W_{1}}{W_{0}}\times \;100\%\;$$

where W_0_ and W_1_ were the weights of the initial and centrifuged samples, respectively.

The swelling behavior of hydrogel and seafood samples was performed by a previous method [27]. The specimens were initially freeze-dried and then weighed (designated as W_d_). Subsequently, they were fully submerged in ultrapure water with a pH of 7 at 25 °C for a duration of 24 h. Following this, the surface moisture was carefully removed, and the swollen samples were weighed and recorded as W_s_. The swelling characteristic was then computed using the following formula:

$$\mathrm{Swelling}\;\mathrm{characteristics}\;=\frac{\;W_{s}\;-\;W_{d}}{W_{d}}\times \;100\%$$

where W_d_ and W_s_ were the weights of the lyophilized and swollen samples, respectively.

### 2.6. Cryogenic Scanning Electron Microscope (Cryo-SEM)

To observe the microstructure, the samples were examined utilizing a cryogenic scanning electron microscope (cryo-SEM, S-4800, Hitachi, Tokyo, Japan) [28]. To preserve the original internal structure, the hydrogel samples were rapidly frozen by immersing the sample holder in liquid nitrogen. After freezing, the hydrogels were fractured within the vacuum chamber to reveal their cross-sectional surfaces. The resulting microstructures were then imaged at an accelerating voltage of 1.0 kV and magnifications of 4.0 k and 10.0 k, respectively.

### 2.7. Microscopes-Fourier Transform Infrared Spectrometer (Micro-FTIR)

The molecular architectures of the specimens were analyzed via a microscope-integrated Fourier transform infrared spectrometer (micro-FTIR, in10 MX, Thermo Ltd., Waltham, MA, USA) in the region of 4000–400 cm^−1^ [28]. The hydrogel samples were freeze-dried for 48 h and data were obtained by averaging 64 scans at a resolution of 2 cm^−1^.

### 2.8. Sensory Assessment

Sensory assessment was conducted on fresh hydrogels and seafood samples (squid and abalone) that had been cooked for 20 min. A panel of twenty assessors (10 males and 10 females) was recruited and told to follow these guidelines of the International Standard method (ISO 8586, 2023). The samples were presented on white paper plates at ambient temperature in a randomized order to each panelist, who evaluated the texture attributes, including hardness, chewiness, springiness, brittleness, and palatability, using a five-level sensory rating scale. On this scale, a rating of 1 represented the minimum level of perceived intensity, while a score of 5 represented the highest. The research was reviewed and approved by the Ethics Committee of Shanghai Jiao Tong University (protocol number: E2021207I), and informed consent forms were provided to all participants.

### 2.9. Statistical Analysis

All experiments were run in triplicate; results are shown as mean ± SD. Significant differences were evaluated using one-way ANOVA (*p* < 0.05) in SPSS 26. Moreover, correlation studies and hierarchical clustering analysis were executed, including heatmap generation, via Origin 22.

## 3. Results and Discussion

### 3.1. Fabrication and Analysis of κ-car-k^+^/KGM Double Networks Hydrogels

#### 3.1.1. Fabrication of κ-car-k^+^/Konjac Glucomannan Double Networks

**Figure 1:**
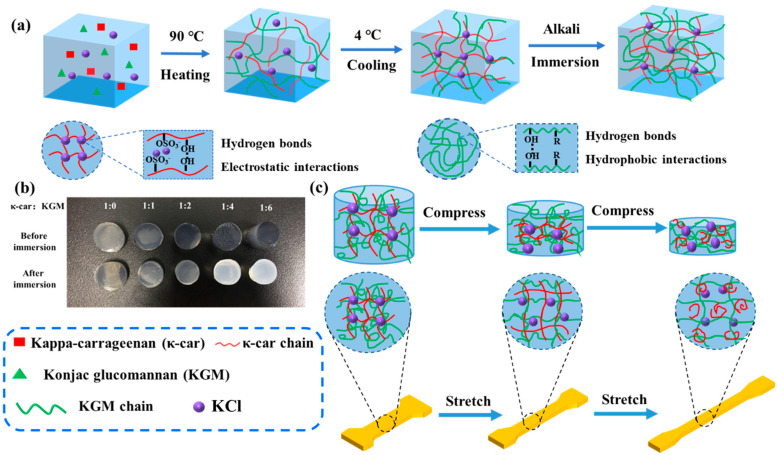
Schematic illustration of the (**a**) preparation process of the κ-car-k^+^/KGM DNs, (**b**) visual changes in the κ-car-k^+^/konjac glucomannan gel system before and after immersion in a 15 wt% Na_2_CO_3_ solution for 24 h at 30 °C, and (**c**) fracture process of the κ-car-k^+^/KGM DNs under compression or tension.

The method for fabricating dual physically crosslinked κ-car-k^+^/konjac glucomannan double networks is illustrated in Figure 1a. In brief, κ-car and KGM powders were dissolved in ultra-pure water, which was subsequently heated at 90 °C under magnetic stirring to obtain a homogeneous solution. Thereafter, KCl granules were added to the aforementioned solution. The resulting solution was then poured into cylindrical molds and stored at 4 °C for 12 h to form hydrogel. During the cooling process, κ-car chains underwent a coil-to-helix transition. The aggregation of single helices into double-helical structures was mediated by potassium ions (K^+^), which acted as ionic bridges. These ions simultaneously formed strong ionic bonds with the negatively charged sulfate groups and established stabilizing ion-dipole interactions with the ether oxygen of the 3,6-anhydro-D-galactose ring on adjacent chains [29]. This process resulted in the formation of the physically linked first-layer κ-car-k^+^ network. Subsequently, the resulting hydrogels, which contained unreacted KGM molecules, were fully submerged in an Na_2_CO_3_ solution to form the second network through hydrogen bonds and hydrophobic interactions due to the deacetylation of KGM molecules [30]. With increasing KGM concentration, the appearance of κ-car-k^+^/KGM DNs was gradually opaque (Figure 1b), the results similar to Hua et al. [31]. The volume and diameter of the samples were slightly reduced, suggesting that the removal of acetyl groups from the KGM molecular chains led to decreased steric hindrance. This facilitated intermolecular interactions and encouraged the development of hydrophobic microdomains, which contributed to network formation [32].

#### 3.1.2. Mechanical Properties Analysis

**Figure 2:**
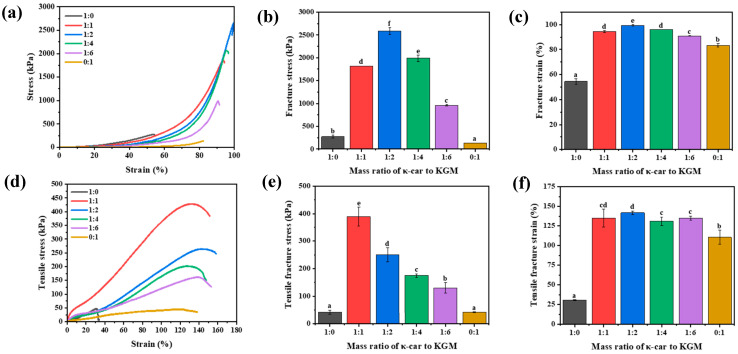
(**a**) Compressive stress-strain curves, (**b**) compressive fracture stress, (**c**) compressive fracture strain, (**d**) tensile stress-strain curves, (**e**) tensile fracture stress, (**f**) tensile fracture strain of the κ-car-k^+^ SNs, KGM SNs, and κ-car-k^+^/konjac glucomannan double networks with mass ratios of 1:1 to 1:6. Different lowercase letters on the bar chart indicates significant differences.

The mechanical performance of hydrogels was systematically assessed through compression and tensile tests to elucidate the synergistic effect of the double network architecture (Figure 1c). Representative compressive stress-strain curves revealed a dramatic enhancement in the mechanical properties of the κ-car-k^+^/KGM DNs compared to their single network (SN) counterparts (Figure 2a). Both κ-car-k^+^ SNs and KGM SNs exhibited brittle and soft characteristics, fracturing at stresses of 272 kPa (strain ~55%) and 133 kPa (strain ~83%), respectively. On the contrary, all DNs displayed typical elastomer-like behavior, withstanding large deformations without failure. The mechanical properties were highly dependent on the KGM content, peaking at a κ-car to KGM mass ratio of 1:2. The optimized κ-car-k^+^/KGM-1:2 DN achieved a remarkable compressive stress of 2587 kPa at 99% strain, which is roughly 10 and 20 times greater than that of the κ-car-k^+^ and KGM SNs, respectively (Figure 2b,c). This substantial improvement indicates the successful construction of a synergistic DN structure where the two networks effectively reinforce each other.

Given that most edible hydrogels are too brittle for tensile evaluation, the tensile properties were further evaluated to highlight the robustness of the prepared DNs (Figure 2d). Consistent with the compression results, all DNs demonstrated significantly improved tensile performance over the SNs. Notably, the κ-car-k^+^/KGM-1:1 DNs presented the highest tensile strength, reaching a fracture stress of ~380 kPa, approximately nine times greater than that of both κ-car-k^+^ and KGM SNs (Figure 2e). Furthermore, its fracture strain reached 144%, far exceeding the limited stretchability of κ-car-k^+^ (31%) and KGM (110%) SNs (Figure 2f). Interestingly, the optimal mass ratio for tensile strength (1:1) differed from that for compressive strength (1:2), suggesting that the load-bearing and energy dissipation mechanisms are sensitive to the mode of deformation. As the KGM content further increased (from 1:2 to 1:6), both tensile and compressive properties gradually declined, likely due to a relative decrease in the proportion of the rigid, energy-dissipating first network.

The exceptional mechanical properties of the κ-car-k^+^/KGM DNs can be attributed to the classic energy dissipation mechanism inherent to DN structures. Upon loading (either compression or tension), the rigid and brittle κ-car-k^+^ first network fractured preferentially. This sacrificial breakdown of its ionic and hydrogen-bonded crosslinks effectively dissipated a large amount of energy, preventing catastrophic failure of the entire structure [33]. Meanwhile, the highly ductile and interconnected KGM second network remained intact, bridging the micro-cracks and sustaining the load, thereby maintaining the hydrogel’s structural integrity and contributing to its high toughness and stretchability [34,35]. To validate this mechanism and optimize the network architecture, the influence of crosslinking parameters on the mechanical characteristics of the κ-car-k^+^/KGM-1:2 DN was investigated (Figure S1). The compressive strength increased from 2032 kPa to 2587 kPa as the KCl concentration rose from 0.2 to 1.0 wt%, confirming that a higher crosslinking density in the rigid first network enhanced the overall gel strength (Figure S1a–c). This is because k^+^ effectively shields electrostatic repulsion and promotes the formation of stable double-helical junctions in the κ-car network [29]. Conversely, the mechanical properties of DNs first increased and then decreased with increasing Na_2_CO_3_ concentration and immersion time, reaching an optimum at 15 wt% Na_2_CO_3_ and 24 h immersion (Figure S1d–i). This indicates that an overly dense second network, potentially caused by excessive dehydration and chain aggregation at high alkaline conditions, can lead to increased brittleness and stress concentration, thereby compromising toughness [32]. Therefore, the superior mechanical performance of the DNs originates from a well-balanced interplay: a densely cross-linked first network provides stiffness and serves as the sacrificial component, while a moderately cross-linked second network ensures ductility and structural integrity. Based on these findings, the optimized conditions (1 wt% KCl, 15 wt% Na_2_CO_3_, 24 h immersion) were used for all subsequent characterizations.

#### 3.1.3. Energy Dissipation and Self-Recovery

**Figure 3:**
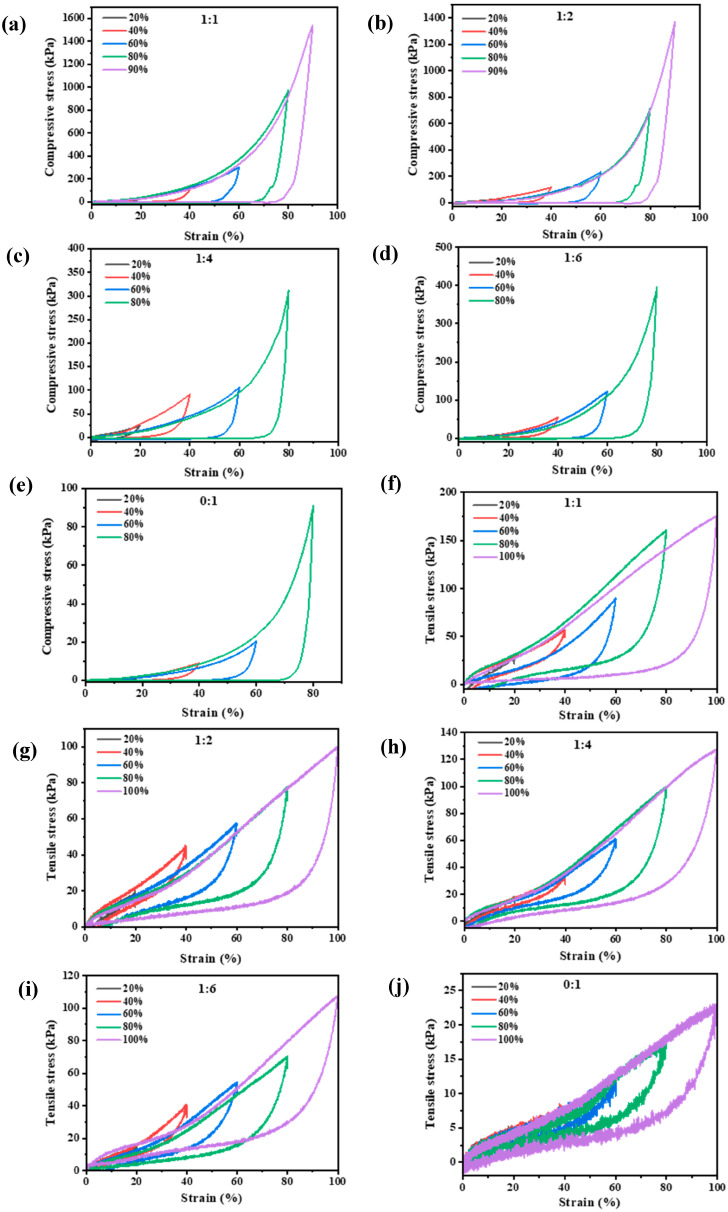
Compressive loading-unloading curves of the (**a**–**d**) κ-car-k^+^/konjac glucomannan double networks with mass ratios of 1:1 to 1:6, and (**e**) KGM SNs; tensile loading-unloading curves of the (**f**–**i**) κ-car-k^+^/konjac glucomannan double networks with mass ratios of 1:1 to 1:6 and (**j**) KGM SNs.

To understand the toughness and resilience of the DNs, we conducted successive cyclic loading-unloading tests in both compression and tension. The energy dissipation capacity (Uhys) is quantified by the area of the hysteresis loop in the first cycle, while the self-recovery efficiency is evaluated by comparing the dissipated energy between the first (U_1_) and second (U_2_) loading cycles [36]. A prominent hysteresis loop was observed for all hydrogels in the first loading-unloading cycle, indicating effective energy dissipation (Figure 3). The area of these loops, representing Uhys, systematically increased with applied strain, which is attributed to the progressive rupture of physical crosslinks within the network [37,38]. In compression, the Uhys of the DNs first increased and then decreased with rising KGM content, with the κ-car-k^+^/KGM-1:2 DN exhibiting the highest energy dissipation at strains up to 40% (Figure S2). In tension, a clearer trend emerged: increasing the proportion of the rigid κ-car network led to a larger hysteresis loop and thus higher Uhys (Figure 3f–j and Figure S3), confirming the primary role of the κ-car network as the main energy-dissipating component.

**Figure 4:**
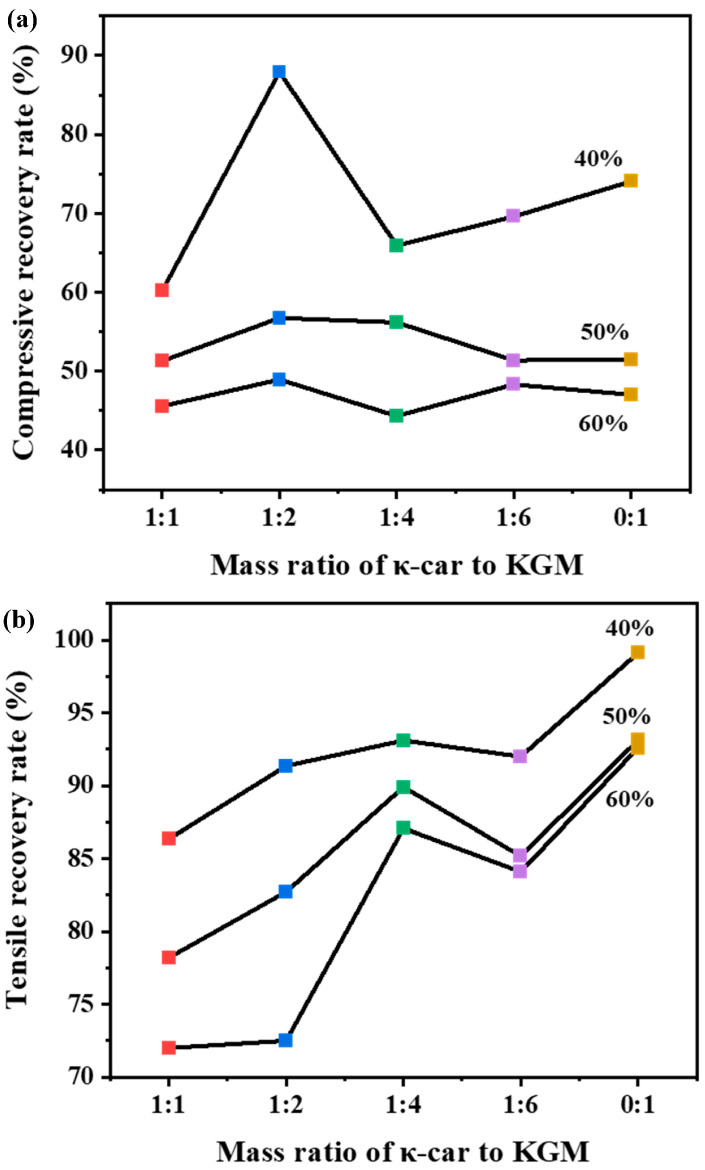
(**a**) Compressive self-recovery capacity and (**b**) tensile self-recovery capacity of the κ-car-k^+^/konjac glucomannan gels with mass ratios of 1:1, 1:2, 1:4, 1:6, and 0:1.

The self-recovery behavior was subsequently analyzed through six consecutive cycles (Figure 4 and Figure S4–S15). After the first cycle, a significant reduction in the hysteresis loop area was observed, indicating that some of the network damage was irreversible on the timescale of the experiment [38]. We quantified the recovery rate (U_2_/U_1_) for different compositions and strains (Figure 4a,b). In compression, the κ-car-k^+^ SN failed during cyclic tests above 50% strain, whereas the DNs remained intact. The κ-car-k^+^/KGM-1:2 DN displayed the best compressive recovery, with a rate of 88% at 40% strain, which decreased to 49% at 60% strain. In tension, the DNs exhibited significantly enhanced self-recovery properties compared to κ-car SNs, although the recovery was lower than that of KGM. Among these DNs, the κ-car-k^+^/KGM-1:4 DN maintained the highest recovery rate, reaching 93% at 40% strain and maintaining 87% even at 60% strain. Moreover, for all DN compositions, the tensile self-recovery rate was significantly higher than the compressive recovery rate at equivalent strains.

This combination of high energy dissipation and excellent self-recovery can be explained by the dynamics of the double network structure. During the initial loading, the rigid κ-car-k^+^ network acted as a sacrificial component. Its abundant and reversible non-covalent crosslinks (ionic bonds and hydrogen bonds) ruptured to dissipate energy, protecting the overall structure from catastrophic failure. Upon unloading, these sacrificial bonds can rapidly reform, enabling the material to recover its mechanical properties [29]. The ductile KGM network provided a continuous, elastic matrix that supported this process and prevented permanent deformation [34]. Therefore, the synergistic interplay between the sacrificial rigid network and the elastic ductile network endows the DNs with their impressive toughness and tunable recovery properties.

#### 3.1.4. Structural and Molecular Characterization

**Figure 5:**
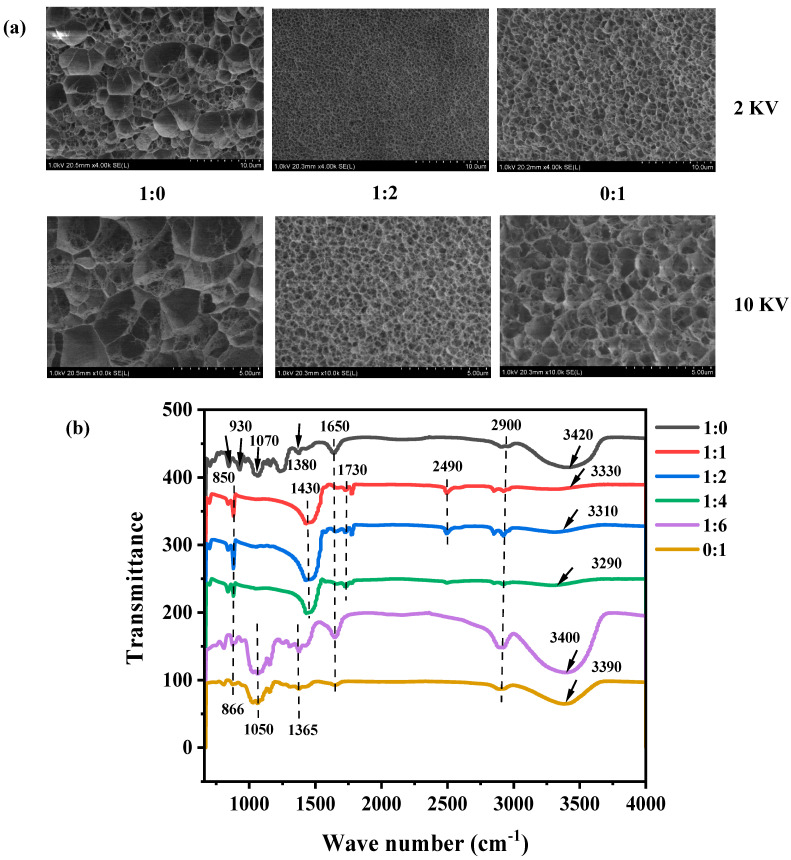
(**a**) Cyro-SEM images, (**b**) Micro-FTIR of the κ-car-k^+^ SNs, KGM SNs, and κ-car-k^+^/konjac glucomannan double networks with mass ratios of 1:1 to 1:6.

To elucidate the structural basis for the enhanced mechanical performance, the microstructure and intermolecular interactions within the hydrogels were investigated using Cryo-SEM and FTIR spectroscopy. Cryo-SEM imaging revealed the three-dimensional porous structures of all hydrogels (Figure 5a). There were significant differences in the microstructure characteristics of the two SNs. The κ-car-k^+^ SNs exhibited a heterogeneous structure with large, irregular pores ranging from 2 to 10 μm. In contrast, the KGM SNs displayed a more uniform network with smaller, evenly distributed pores (~1.5–2 μm). Notably, the κ-car-k^+^/KGM-1:2 DNs exhibited a remarkably different microstructure: it was significantly denser and more homogeneous, characterized by much smaller and well-defined pores with an average size of ~0.5 μm. This compact and uniform network structure, resulting from greater entanglement between the two polymer networks, facilitated more effective stress distribution and effectively hindered crack propagation [39]. This provided a clear microstructural explanation for the substantial improvements in strength and toughness observed in the DNs.

To understand the molecular structure of the hydrogels, micro-FTIR analysis was conducted, and the results were shown in Figure 5b. First, the spectrum of the KGM SNs confirmed the successful deacetylation during the alkaline treatment, as evidenced by the disappearance of the characteristic C=O stretching peak of acetyl groups at 1730 cm^−1^, which was present in native KGM powder [40]. Importantly, no new adsorption peaks were observed in the FTIR spectra of κ-car-k^+^/KGM DNs compared to their individual components. This absence of new peaks confirms that the DN formation is governed by physical interactions rather than the creation of new covalent chemical bonds, which is consistent with our design strategy (physical DNs). Moreover, a distinct redshift of the broad O-H peak from approximately 3420 cm^−1^ in the κ-car-k^+^ SNs or 3390 cm^−1^ in the KGM SNs to 3290 cm^−1^ in the DN was observed. This significant shift is indicative of the formation of stronger and/or more numerous intermolecular hydrogen bonds between the hydroxyl groups of the κ-car and KGM polymer chains [29]. In summary, the combined structural and spectroscopic analyses provide evidence that the synergy between the κ-car and KGM networks leads to a dense, physically interlocked microstructure. This structure is stabilized by enhanced intermolecular hydrogen bonding, which is the fundamental reason for the remarkable mechanical performance of the DNs.

### 3.2. Application as Biomimetic Seafood Analogues

#### 3.2.1. Mechanical and Textural Characteristics

**Figure 6:**
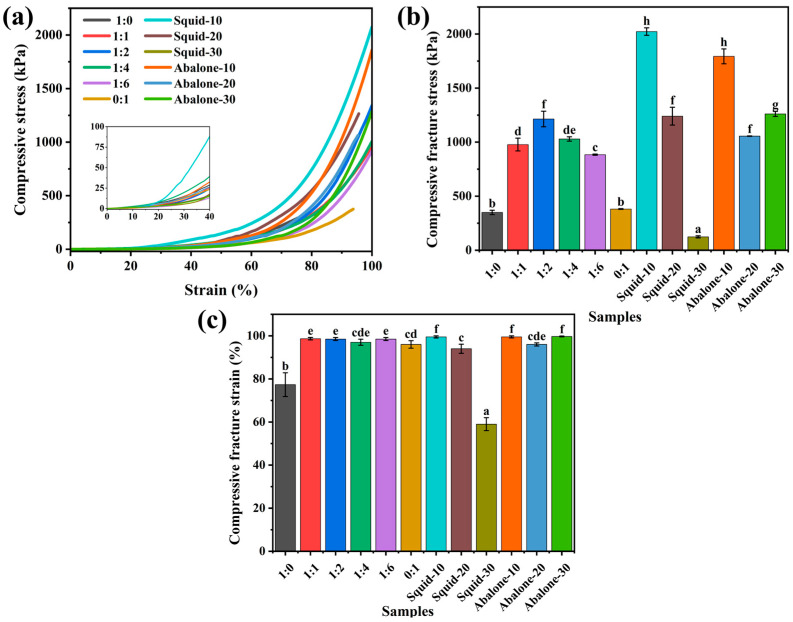
(**a**) Compressive stress-strain profiles, (**b**) fracture stress, and (**c**) fracture strain of the κ-car-k^+^ SNs, KGM SNs, and κ-car-k^+^/konjac glucomannan double networks with mass ratios of 1:1 to 1:6, and squid and abalone cooked for 10, 20, and 30 min, respectively. Different lowercase letters on the bar chart indicates significant differences.

**Figure 7:**
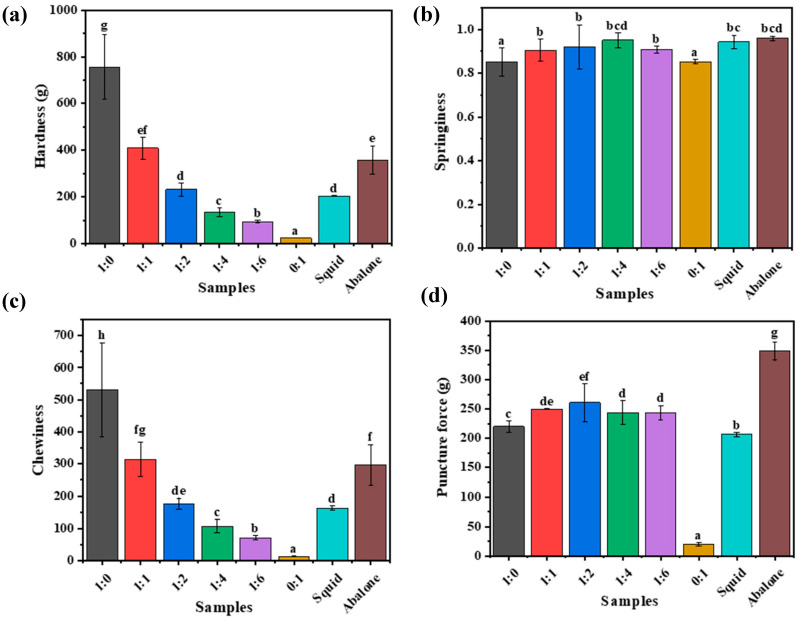
(**a**) Hardness, (**b**) springiness, (**c**) chewiness, (**d**) puncture force of the κ-car-k^+^ SNs, KGM SNs, κ-car-k^+^/konjac glucomannan double networks with mass ratios of 1:1 to 1:6, and squid and abalone cooked for 20 min, respectively. Different lowercase letters on the bar chart indicates significant differences.

To investigate the potential of the κ-car-k^+^/KGM DNs as seafood analogues with high chewiness, their physical properties were systematically compared to those of two popular seafood products known for their characteristic textures: abalone and squid. As illustrated in Figure 6a–c, a moderate increase in KGM concentration led to improved compressive mechanical properties of the κ-car-k^+^/KGM DNs. When the κ-car to KGM mass ratio was adjusted to 1:2, the DNs exhibited higher gel strength and toughness, which were comparable to those of squid cooked for 20 min. Meanwhile, the κ-car-k^+^/KGM-1:1 DNs showed similar mechanical performance to abalone also cooked for 20 min. Subsequently, the texture profile analysis (TPA) of both squid and abalone cooked for 20 min was conducted for further comparison (Figure 7a–d). Elevating the KGM concentration resulted in a reduction in both hardness and chewiness of the κ-car-k^+^/KGM gels, whereas moderate increases in KGM concentration enhanced their springiness and puncture force [41]. Compared to cooked squid and abalone, the κ-car-k^+^/KGM-1:1 DNs and κ-car-k^+^/KGM-1:2 DNs exhibited comparable hardness, springiness, and chewiness to abalone and squid, respectively, although differences were observed in puncture force. Overall, these findings indicate that the κ-car-k^+^/KGM-1:1 DNs and κ-car-k^+^/KGM-1:2 DNs have the potential to mimic the mechanical behavior of abalone and squid, respectively.

#### 3.2.2. Water Retention Ability and Swelling Characteristics

**Figure 8:**
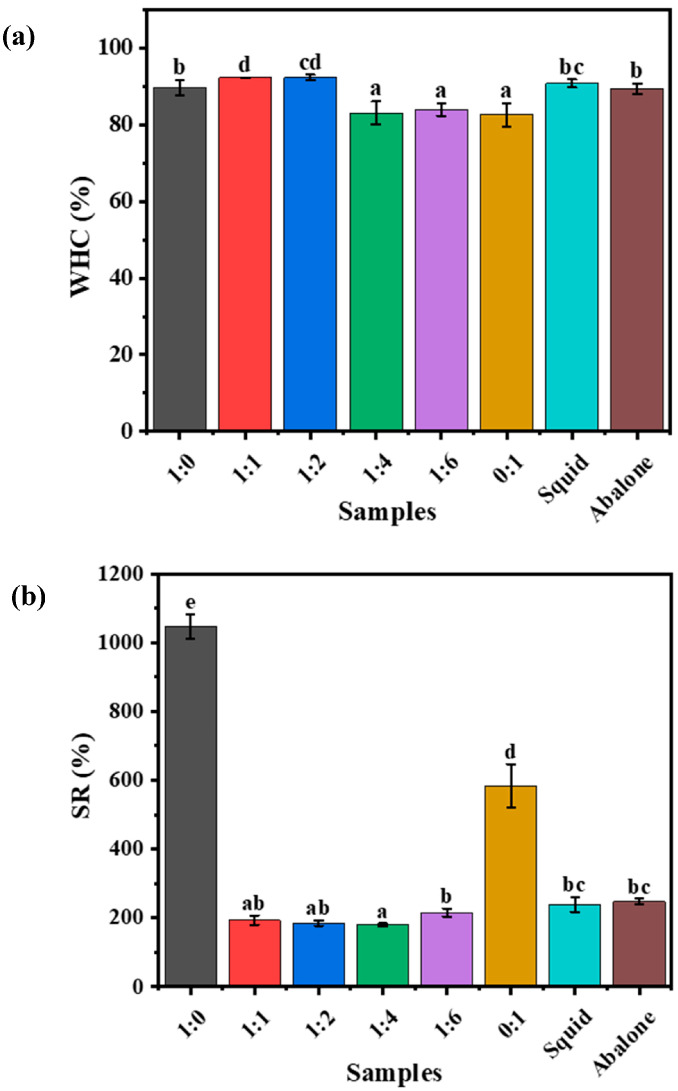
(**a**) WHC, (**b**) SR of the κ-car-k^+^ SNs, KGM SNs, κ-car-k^+^/KGM DNs with mass ratios ranging from 1:1 to 1:6, and squid and abalone cooked for 20 min, respectively. Different lowercase letters on the bar chart indicates significant differences.

A high water-holding capacity (WHC) is advantageous for minimizing water loss, preserving freshness, and enhancing the texture of food products by providing a resilient and firm mouthfeel [3]. Therefore, the WHC of hydrogels and seafoods was evaluated. As illustrated in Figure 8a, all hydrogel samples exhibited a high water-retention ability (>80%). With increasing konjac glucomannan concentration, the water-retention ability of the κ-car-k^+^/konjac glucomannan gels initially increased and then decreased. The κ-car-k^+^/KGM-1:1 DNs and the κ-car-k^+^/KGM-1:2 DNs exhibited the higher WHC. This improvement could be attributed to their denser microstructures and enhanced intermolecular interactions (Figure 5). Further increasing KGM concentration led to a decrease in WHC, likely due to the formation of more hydrophobic micro-domains [42]. In addition, when the mass ratio of κ-car to KGM was 1:0–1:2, the DNs showed WHC values comparable to those of squid and abalone.

Swelling behavior refers to the volume expansion of macromolecules in a solvent, and the swelling capacity of hydrogels is typically quantified by the SR. As shown in Figure 8b, there was no significant SR difference among the various κ-car-k^+^/KGM DNs, which was significantly lower than that of the κ-car-k^+^ and KGM SNs, respectively. This was due to their denser microstructures and stronger intermolecular interactions (Figure 5). During the swelling process, water penetrated the hydrogel matrix and expanded the volume of SNs; however, the tightly crosslinked structure of DNs tended to contract, limiting further water uptake. This shrinkage effect resulted in a lower SR compared to SNs [43]. Additionally, the κ-car-k^+^/KGM-1:1 DNs and the κ-car-k^+^/KGM-1:2 DNs presented the comparable SR with abalone and squid, respectively, consistent with their WHC trends. Therefore, these two DN formulations demonstrate great potential for mimicking the mouthfeel of abalone and squid based on the results of water retention ability and swelling characteristics.

#### 3.2.3. Analogical Analysis of κ-car-k^+^/KGM Gels and Seafood in Statistics

##### Correlation Analysis

**Figure 9:**
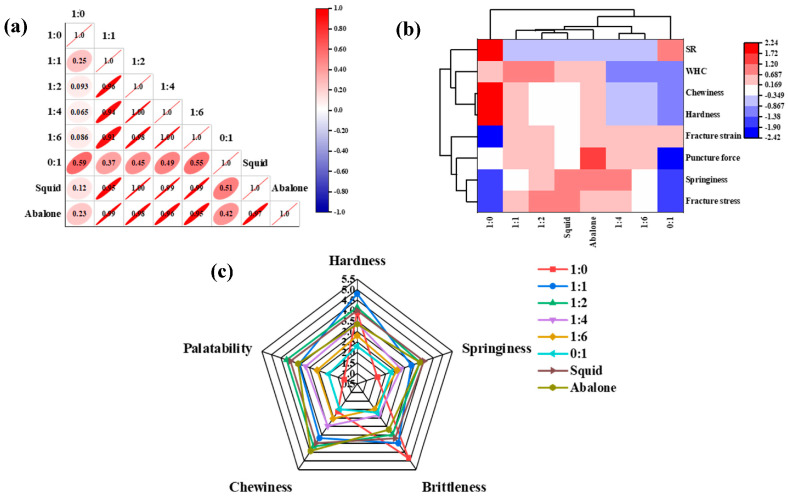
(**a**) Radar chart of the κ-car-k^+^/konjac glucomannan gels with mass ratios of 1:0, 1:1, 1:2, 1:4, 1:6, and 0:1 and seafood samples (squid and abalone) on the sensory attributes (hardness, springiness, chewiness, brittleness, and palatability); (**b**) correlation analysis; (**c**) HCA heatmap analysis of the κ-car-k^+^/konjac glucomannan gels with mass ratios of 1:0, 1:1, 1:2, 1:4, 1:6, and 0:1 and seafood samples (squid and abalone) on the physical properties (compressive fracture stress, compressive fracture strain, hardness, springiness, chewiness, puncture force, WHC, and SR).

To further investigate the relationship between the hydrogel formulations and seafood samples, a comprehensive correlation analysis was carried out using the measured parameters, including fracture stress, fracture strain, hardness, springiness, chewiness, puncture force, water-holding capacity (WHC), and swelling ratio (SR). As illustrated in Figure 9a, each rectangular box contained ellipses with varying shapes and colors representing the correlation coefficients, where a color gradient from blue to red indicated a transition from negative to positive correlation. The correlation plot showed that the entire series of κ-car-k^+^/KGM DNs (ratios 1:1 to 1:6) demonstrated high correlation coefficients with both abalone (r = 0.95–0.99) and squid (r = 0.95–1.00), confirming the overall suitability of the DN system for mimicking these textures. Most notably, the κ-car-k^+^/KGM-1:1 DNs and κ-car-k^+^/KGM-1:2 DNs exhibited the highest association (correlation coefficient of 0.99 and 1.00) with abalone and squid, respectively.

##### Hierarchical Cluster Associated with Heatmap (HCA Heatmap) Analysis

The relationship of hydrogels and seafood samples was also plotted using HCA heatmap analysis. As shown in Figure 9b, data values corresponded with different colors in each rectangular tiling, where blue to red color presented low to high abundance. The analysis clearly classified the eight samples into four distinct clusters. Significantly, in cluster I, grouped the κ-car-k^+^/KGM-1:1 and -1:2 DNs together with the natural abalone and squid samples, all of which were characterized by low swelling ratios and superior mechanical and textural properties. A closer examination within this cluster revealed that the κ-car-k^+^/KGM-1:1 DNs shared five key parameters with abalone, making it a stronger match than to squid (one shared parameter). Conversely, the κ-car-k^+^/KGM-1:2 DNs aligned more closely with squid, sharing four key parameters. Therefore, the HCA provided strong visual support for the conclusion that the κ-car-k^+^/KGM-1:1 DN most closely resembled abalone, particularly in fracture stress, fracture strain, hardness, chewiness, and swelling ratio, while the κ-car-k^+^/KGM-1:2 DN was the best mimic for squid.

#### 3.2.4. Sensory Assessment Analysis

The sensory attributes of the hydrogels and seafood samples were assessed (Figure 9c). Prior to sensory testing, the prepared gels were immersed in water to eliminate residual Na_2_CO_3_ until the pH of the water reached neutrality. This ensured that the participants’ sensory perception was not influenced by any alkaline flavor. As the KGM content increased in the DNs, perceived hardness and brittleness decreased, while elasticity, chewiness, and palatability first increased and then declined. The κ-car-k^+^/KGM-1:2 DNs received the highest scores for elasticity, chewiness, and palatability, outperforming all other hydrogel formulations. Crucially, when benchmarked against the natural seafood, the sensory profile of the κ-car-k^+^/KGM-1:2 DNs was remarkably similar to that of squid across all five evaluated attributes (hardness, elasticity, chewiness, brittleness, and palatability). Similarly, the κ-car-k^+^/KGM-1:1 DNs closely matched the sensory profile of abalone, particularly in elasticity, chewiness, brittleness, and palatability. These results provide the ultimate validation that the prepared DNs are not only instrumentally comparable but are also perceived by humans as having textures highly analogous to squid and abalone.

## 4. Conclusions

In this study, the κ-car-k^+^/KGM DNs were innovatively fabricated using a single heating-cooling cycle combined with immersion in a Na_2_CO_3_ solution. The enhanced crosslinking density of the rigid network and the appropriately adjusted crosslinking density of the ductile network contributed to significantly improved mechanical performance in the κ-car-k^+^/KGM DNs. Specifically, the κ-car-k^+^/KGM-1:2 DNs exhibited exceptional strength and toughness under both compressive and tensile loading, surpassing the mechanical properties of both κ-car-k^+^ SNs and KGM SNs. This enhancement was primarily attributed to the sequential deformation mechanism: the rigid network fractured first, dissipating energy, while the ductile KGM network remained intact, effectively maintaining the structural integrity of the hydrogel. Moreover, these hydrogels demonstrated superior tensile self-recovery capacity compared to compressive recovery. When compared to natural seafood samples, the κ-car-k^+^/KGM-1:1 and κ-car-k^+^/KGM-1:2 DNs exhibited comparable mechanical properties, textural characteristics, WHC, SR, and sensory attributes to abalone and squid, respectively, indicating their promising potential as biomimetic seafood products with high chewiness. This comprehensive demonstration establishes a robust framework and proof-of-concept for engineering complex textural properties in plant-based alternatives. Building upon this foundational proof-of-concept, future research will focus on integrating comprehensive nutritional and sensory profiles to broaden the applicability of this versatile platform. Furthermore, addressing critical aspects for practical application, such as long-term stability, raw material variability, and industrial scalability, will be crucial for translating this promising technology from the laboratory to the market.

## Data Availability

The original contributions presented in the study are included in the article/Supplementary Material, further inquiries can be directed to the corresponding author.

## Supplementary Materials

The following supporting information can be downloaded at: https://www.mdpi.com/article/10.3390/foods14173140/s1, Figure S1: (a) Compressive stress-strain curves, (b) fracture stress, (c) fracture strain of the κ-car-k^+^/KGM-1:2 DNs with different KCl concentration (0.2–1.0 wt%), (d) compressive stress-strain curves, (e) fracture stress, (f) fracture strain of the κ-car-k^+^/KGM-1:2 DNs with different immersed Na_2_CO_3_ concentration (5–25 wt%), (g) compressive stress-strain curves, (h) fracture stress, (i) fracture strain of the κ-car-k^+^/KGM-1:2 DNs with different immersion time (6–30 h) of Na_2_CO_3_ solution; Figure S2: The compressive U_hys_ of the κ-car-k^+^/KGM gels with different mass ratio at (a) 20%, (b) 40%, (c) 60%, (d) 80% strain; Figure S3: The tensile U_hys_ of the κ-car-k^+^/KGM gels with different mass ratio at (a) 20%, (b) 40%, (c) 60%, (d) 80%, (e) 100% strain; Figure S4: The cyclic compressive loading-unloading curves of (a) κ-car-k^+^ SNs, (b–e) κ-car-k^+^/KGM DNs with mass ratio of 1:1 to 1:6, (f) KGM SNs with 6 times loading number at 40% strain; Figure S5: The compressive U_hys_ of (a) κ-car-k^+^ SNs, (b–e) κ-car-k^+^/KGM DNs with mass ratio of 1:1 to 1:6, (f) KGM SNs with 6 times loading number at 40% strain, (g) compressive recovery rate of κ-car-k^+^/KGM gels at 40% strain; Figure S6: The cyclic compressive loading-unloading curves of (a) κ-car-k^+^ SNs, (b–e) κ-car-k^+^/KGM DNs with mass ratio of 1:1 to 1:6, (f) KGM SNs with 6 times loading number at 50% strain; Figure S7: The compressive U_hys_ of (a–d) κ-car-k^+^/KGM DNs with mass ratio of 1:1 to 1:6, (e) KGM SNs with 6 times loading number at 50% strain, (f) compressive recovery rate of κ-car-k^+^/KGM gels at 50% strain; Figure S8: The cyclic compressive loading-unloading curves of (a) κ-car-k^+^ SNs, (b–e) κ-car-k^+^/KGM DNs with mass ratio of 1:1 to 1:6, (f) KGM SNs with 6 times loading number at 60% strain; Figure S9: The compressive U_hys_ of (a–d) κ-car-k^+^/KGM DNs with mass ratio of 1:1 to 1:6, (e) KGM SNs with 6 times loading number at 60% strain, (f) compressive recovery rate of κ-car-k^+^/KGM gels at 60% strain; Figure S10: The cyclic tensile loading-unloading curves of (a–d) κ-car-k^+^/KGM DNs with mass ratio of 1:1 to 1:6, (e) KGM SNs with 6 times loading number at 40% strain; Figure S11: The tensile U_hys_ of (a–d) κ-car-k^+^/KGM DNs with mass ratio of 1:1 to 1:6, (e) KGM SNs with 6 times loading number at 40% strain, (f) tensile recovery rate of κ-car-k^+^/KGM gels at 40% strain; Figure S12: The cyclic tensile loading-unloading curves of (a–d) κ-car-k^+^/KGM DNs with mass ratio of 1:1 to 1:6, (e) KGM SNs with 6 times loading number at 50% strain; Figure S13: The tensile U_hys_ of (a–d) κ-car-k^+^/KGM DNs with mass ratio of 1:1 to 1:6, (e) KGM SNs with 6 times loading number at 50% strain, (f) tensile recovery rate of κ-car-k^+^/KGM gels at 50% strain; Figure S14: The cyclic tensile loading-unloading curves of (a–d) κ-car-k^+^/KGM DNs with mass ratio of 1:1 to 1:6, (e) KGM SNs with 6 times loading number at 60% strain; Figure S15: The tensile U_hys_ of (a–d) κ-car-k^+^/KGM DNs with mass ratio of 1:1 to 1:6, (e) KGM SNs with 6 times loading number at 60% strain, (f) tensile recovery rate of κ-car-k^+^/KGM gels at 60% strain.

## References

[1] Zeng S., Zhang J., Zu G., Huang J. (2021). Transparent, flexible, and multifunctional starch-based double-network hydrogels as high-performance wearable electronics. Carbohydr. Polym..

[2] Su C.-Y., Li D., Wang L.-J. (2025). From micropores to mechanical strength: Fabrication and characterization of edible corn starch-sodium alginate double network hydrogels with Ca^2+^ cross-linking. Food Chem..

[3] Ahmad S., Ahmad M., Manzoor K., Purwar R., Ikram S. (2019). A review on latest innovations in natural gums based hydrogels: Preparations & applications. Int. J. Biol. Macromol..

[4] Gong J.P. (2010). Why are double network hydrogels so tough?. Soft Matter.

[5] Du M., Zhao Y., Zhang Y., Sun S., Fang Y. (2022). Fabrication of agarose/fish gelatin double-network hydrogels with high strength and toughness for the development of artificial beef tendons. Food Funct..

[6] Chen F., Chen Q., Zhu L., Tang Z., Li Q., Qin G., Yang J., Zhang Y., Ren B., Zheng J. (2018). General Strategy To Fabricate Strong and Tough Low-Molecular-Weight Gelator-Based Supramolecular Hydrogels with Double Network Structure. Chem. Mater..

[7] Tang Z., Lyu X., Xiao A., Shen Z., Fan X. (2018). High-Performance Double-Network Ion Gels with Fast Thermal Healing Capability via Dynamic Covalent Bonds. Chem. Mater..

[8] Hou J.-J., Guo J., Wang J.-M., He X.-T., Yuan Y., Yin S.-W., Yang X.-Q. (2015). Edible double-network gels based on soy protein and sugar beet pectin with hierarchical microstructure. Food Hydrocoll..

[9] Sun J., Ren F., Chang Y., Wang P., Li Y., Zhang H., Luo J. (2018). Formation and structural properties of acid-induced casein–agar double networks: Role of gelation sequence. Food Hydrocoll..

[10] Wang Y., Jiao A., Qiu C., Liu Q., Yang Y., Bian S., Zeng F., Jin Z. (2022). A combined enzymatic and ionic cross-linking strategy for pea protein/sodium alginate double-network hydrogel with excellent mechanical properties and freeze-thaw stability. Food Hydrocoll..

[11] Yiu C.C.Y., Wang Y., Selomulya C. (2025). Double Network as a Design Paradigm for Structuring Emulsion Gels in Food. Compr. Rev. Food Sci. Food Saf..

[12] Chen H., Gan J., Ji A., Song S., Yin L. (2019). Development of double network gels based on soy protein isolate and sugar beet pectin induced by thermal treatment and laccase catalysis. Food Chem..

[13] Yan W., Zhang B., Yadav M.P., Feng L., Yan J., Jia X., Yin L. (2020). Corn fiber gum-soybean protein isolate double network hydrogel as oral delivery vehicles for thermosensitive bioactive compounds. Food Hydrocoll..

[14] Du M., Lu W., Zhang Y., Mata A., Fang Y. (2021). Natural polymer-sourced interpenetrating network hydrogels: Fabrication, properties, mechanism and food applications. Trends Food Sci. Technol..

[15] Gong J., Wang L., Wu J., Yuan Y., Mu R.-J., Du Y., Wu C., Pang J. (2019). The rheological and physicochemical properties of a novel thermosensitive hydrogel based on konjac glucomannan/gum tragacanth. LWT.

[16] Sun Y., Xu X., Zhang Q., Zhang D., Xie X., Zhou H., Wu Z., Liu R., Pang J. (2023). Review of Konjac Glucomannan Structure, Properties, Gelation Mechanism, and Application in Medical Biology. Polymers.

[17] Patel A.K., Vadrale A.P., Singhania R.R., Michaud P., Pandey A., Chen S.-J., Chen C.-W., Dong C.-D. (2023). Algal polysaccharides: Current status and future prospects. Phytochem. Rev..

[18] Heidarian P., Kouzani A.Z., Kaynak A., Paulino M., Nasri-Nasrabadi B., Zolfagharian A., Varley R. (2020). Dynamic plant-derived polysaccharide-based hydrogels. Carbohydr. Polym..

[19] Liu Z., Sun C., Hu S., Zhao G., Zhou Y. (2025). A fracture mechanics approach to investigating the crunchy texture of konjac glucomannan gels through imitative chewing tests. Food Hydrocoll..

[20] Lin H.-T.V., Wu H.-X., Sung W.-C. (2021). Hardness and quality of abalone (Haliotis discus hannai diversicolor diversicolor) muscle as suitably softened for seniors. Int. J. Food Prop..

[21] Yu M.-M., Li D.-Y., Liu Z.-Q., Liu Y.-X., Zhou J.-Z., Zhang M., Zhou D.-Y., Zhu B.-W. (2021). Effects of heat treatments on texture of abalone muscles and its mechanism. Food Biosci..

[22] Wang J., Xu Z., Lu W., Zhou X., Liu S., Zhu S., Ding Y. (2024). Improving the texture attributes of squid meat (*Sthenoteuthis oualaniensis*) with slight oxidative and phosphate curing treatments. Food Res. Int..

[23] Chen F., Tang Z., Lu S., Zhu L., Wang Q., Gang Q., Yang J., Chen Q. (2019). Fabrication and mechanical behaviors of novel supramolecular/polymer hybrid double network hydrogels. Polymer.

[24] Fatehi F., Krizsan S.J., Gidlund H., Huhtanen P. (2015). A comparison of ruminal or reticular digesta sampling as an alternative to sampling from the omasum of lactating dairy cows. J. Dairy Sci..

[25] Sow L.C., Tan S.J., Yang H. (2019). Rheological properties and structure modification in liquid and gel of tilapia skin gelatin by the addition of low acyl gellan. Food Hydrocoll..

[26] Zhao H., Chen J., Hemar Y., Cui B. (2020). Improvement of the rheological and textural properties of calcium sulfate-induced soy protein isolate gels by the incorporation of different polysaccharides. Food Chem..

[27] Xu C., Zhan W., Tang X., Mo F., Fu L., Lin B. (2018). Self-healing chitosan/vanillin hydrogels based on Schiff-base bond/hydrogen bond hybrid linkages. Polym. Test..

[28] Chen H., Shi P., Fan F., Chen H., Wu C., Xu X., Wang Z., Du M. (2020). Hofmeister effect-assisted one step fabrication of fish gelatin hydrogels. LWT.

[29] Deng Y., Huang M., Sun D., Hou Y., Li Y., Dong T., Wang X., Zhang L., Yang W. (2018). Dual Physically Cross-Linked κ-Carrageenan-Based Double Network Hydrogels with Superior Self-Healing Performance for Biomedical Application. ACS Appl. Mater. Interfaces.

[30] Kanniyappan H., Thangavel P., Chakraborty S., Arige V., Muthuvijayan V. (2020). Design and evaluation of Konjac glucomannan-based bioactive interpenetrating network (IPN) scaffolds for engineering vascularized bone tissues. Int. J. Biol. Macromol..

[31] Hua J., Liu C., Ng P.F., Fei B. (2021). Bacterial cellulose reinforced double-network hydrogels for shape memory strand. Carbohydr. Polym..

[32] Ji L., Xue Y., Zhang T., Li Z., Xue C. (2017). The effects of microwave processing on the structure and various quality parameters of Alaska pollock surimi protein-polysaccharide gels. Food Hydrocoll..

[33] Weiss J., Salminen H., Moll P., Schmitt C. (2019). Use of molecular interactions and mesoscopic scale transitions to modulate protein-polysaccharide structures. Adv. Colloid Interface Sci..

[34] Hua M., Wu S., Ma Y., Zhao Y., Chen Z., Frenkel I., Strzalka J., Zhou H., Zhu X., He X. (2021). Strong tough hydrogels via the synergy of freeze-casting and salting out. Nature.

[35] Zhu S., Wang Y., Ding Y., Xiang X., Yang Q., Wei Z., Song H., Liu S., Zhou X. (2024). Improved texture properties and toughening mechanisms of surimi gels by double network strategies. Food Hydrocoll..

[36] Wang J.J., Wang Y., Wang Q., Yang J., Hu S.-Q., Chen L. (2019). Mechanically Strong and Highly Tough Prolamin Protein Hydrogels Designed from Double-Cross-Linked Assembled Networks. ACS Appl. Polym. Mater..

[37] Liu S., Li L. (2016). Recoverable and Self-Healing Double Network Hydrogel Based on κ-Carrageenan. ACS Appl. Mater. Interfaces.

[38] Shang X., Wang Q., Li J., Zhang G., Zhang J., Liu P., Wang L. (2021). Double-network hydrogels with superior self-healing properties using starch reinforcing strategy. Carbohydr. Polym..

[39] Zhu Y., Wang Y., Zhang L., Chen X., Dong Y., Zou L., Liu W. (2025). Engineering soy protein/konjac glucomannan double network hydrogels with low voltage-electric field: Anisotropic structure and properties. Food Res. Int..

[40] Chen Y., Zhao H., Liu X., Li Z., Liu B., Wu J., Shi M., Norde W., Li Y. (2016). TEMPO-oxidized Konjac glucomannan as appliance for the preparation of hard capsules. Carbohydr. Polym..

[41] Ma S., Youssef M., Albahi A., Li J., Zhou P., Li B. (2025). Calcium alginate-cross-linked deacetylated konjac glucomannan-based double network hydrogels: Construction, characterizations and gelation kinetics. Int. J. Biol. Macromol..

[42] Chu L., Yang L., Li J., Lin L., Zheng G. (2019). Effect of Smilax china L. starch on the gel properties and interactions of calcium sulfate-induced soy protein isolate gel. Int. J. Biol. Macromol..

[43] Babaei J., Mohammadian M., Madadlou A. (2019). Gelatin as texture modifier and porogen in egg white hydrogel. Food Chem..

