# Supplementary material for: Engineering Edible Double Network Hydrogels with Abalone- and Squid-like Textures from Carrageenan and Konjac Glucomannan
Source: Foods. 2025 Sep 8;14(17):3140. doi: 10.3390/foods14173140 (PMC12428326; doi:10.3390/foods14173140)
Supplement: Supplementary file 1 [file foods-14-03140-s001.zip › foods-3839969-supplementary.pdf]

Supplementary information

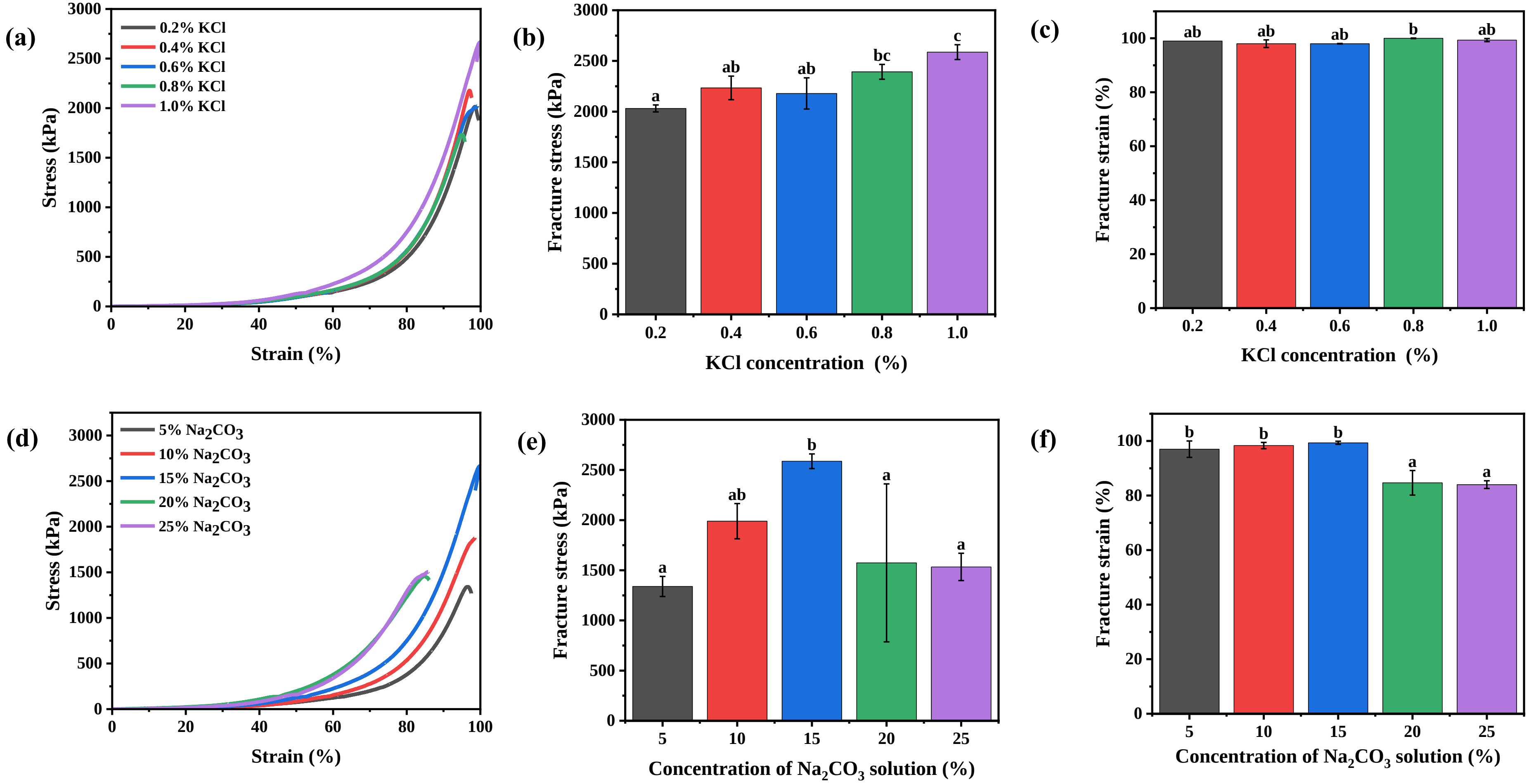

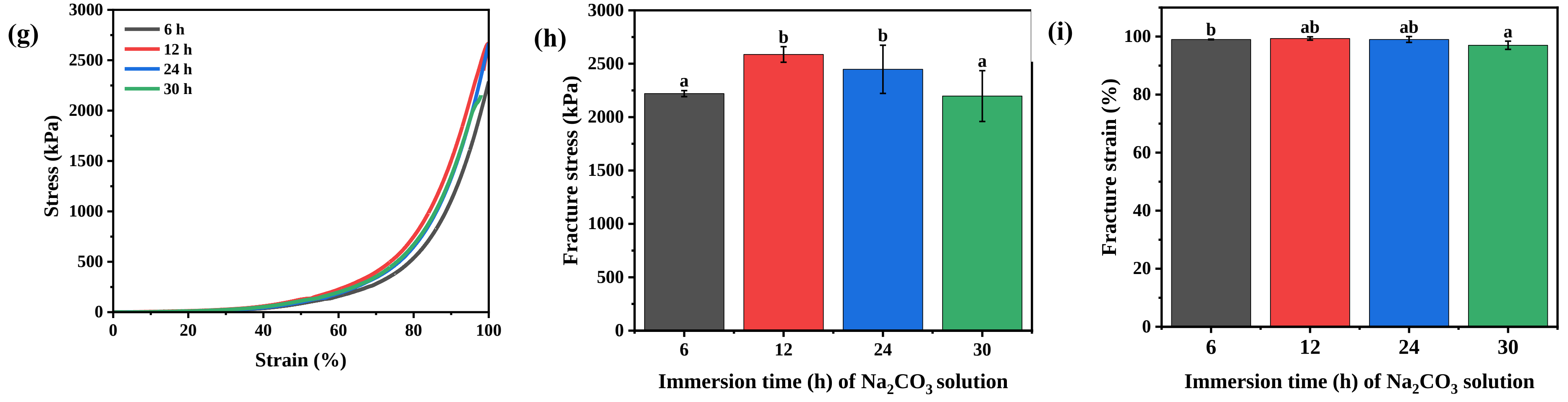

Fig.S1. (a) Compressive stress-strain curves, (b) fracture stress, (c) fracture strain of the κ-car-k$^+$/KGM-1:2 DNs with different KCl concentration (0.2-1.0 wt%); (d) compressive stress-strain curves, (e) fracture stress, (f) fracture strain of the κ-car-k$^+$/KGM-1:2 DNs with different immersed Na_2CO_3 concentration (5-25 wt%); (g) compressive stress-strain curves, (h) fracture stress, (i) fracture strain of the κ-car-k$^+$/KGM-1:2 DNs with different immersion time (6-30 h) of Na_2CO_3 solution.

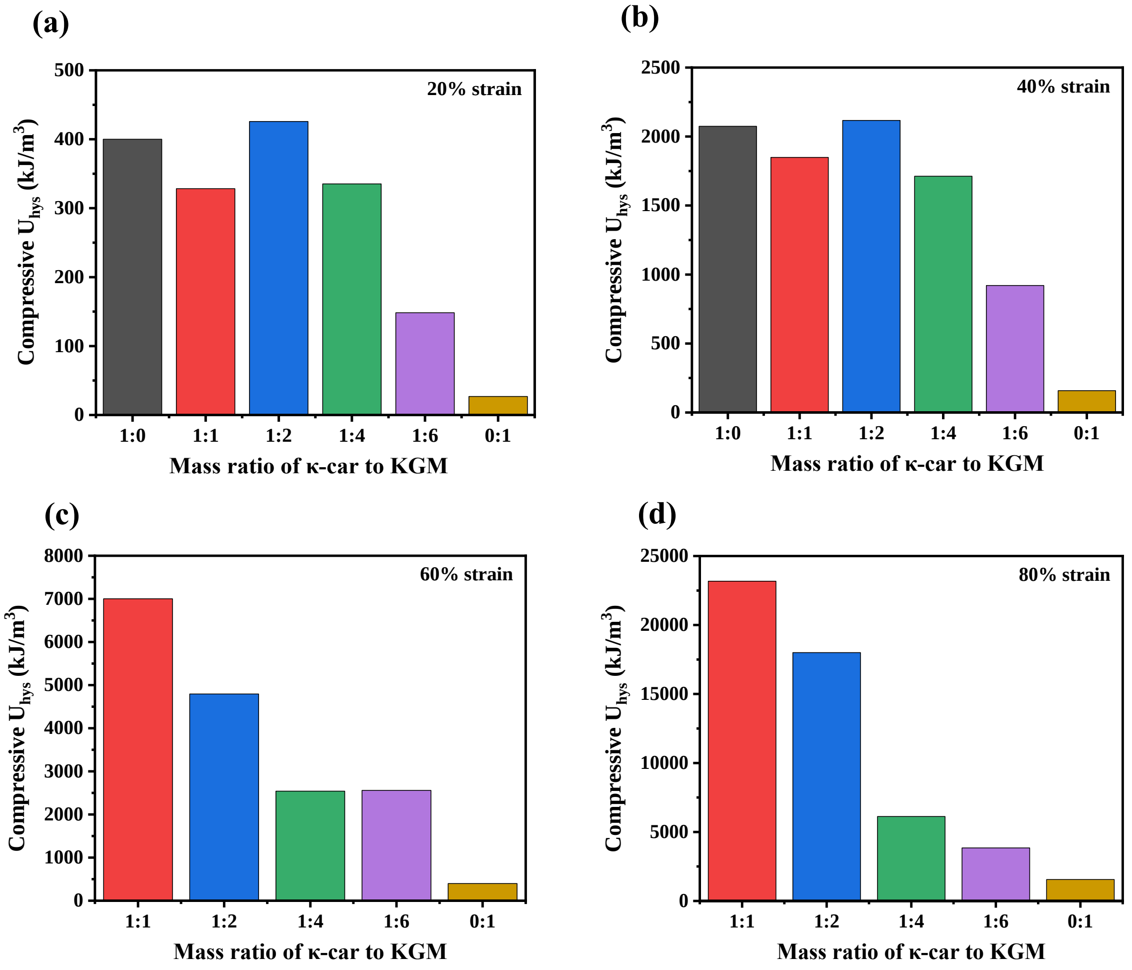

Fig. S2. The compressive U_{hys} of the κ-car-k^+/KGM gels with different mass ratio at (a) 20%, (b) 40%, (c) 60%, (d) 80% strain.

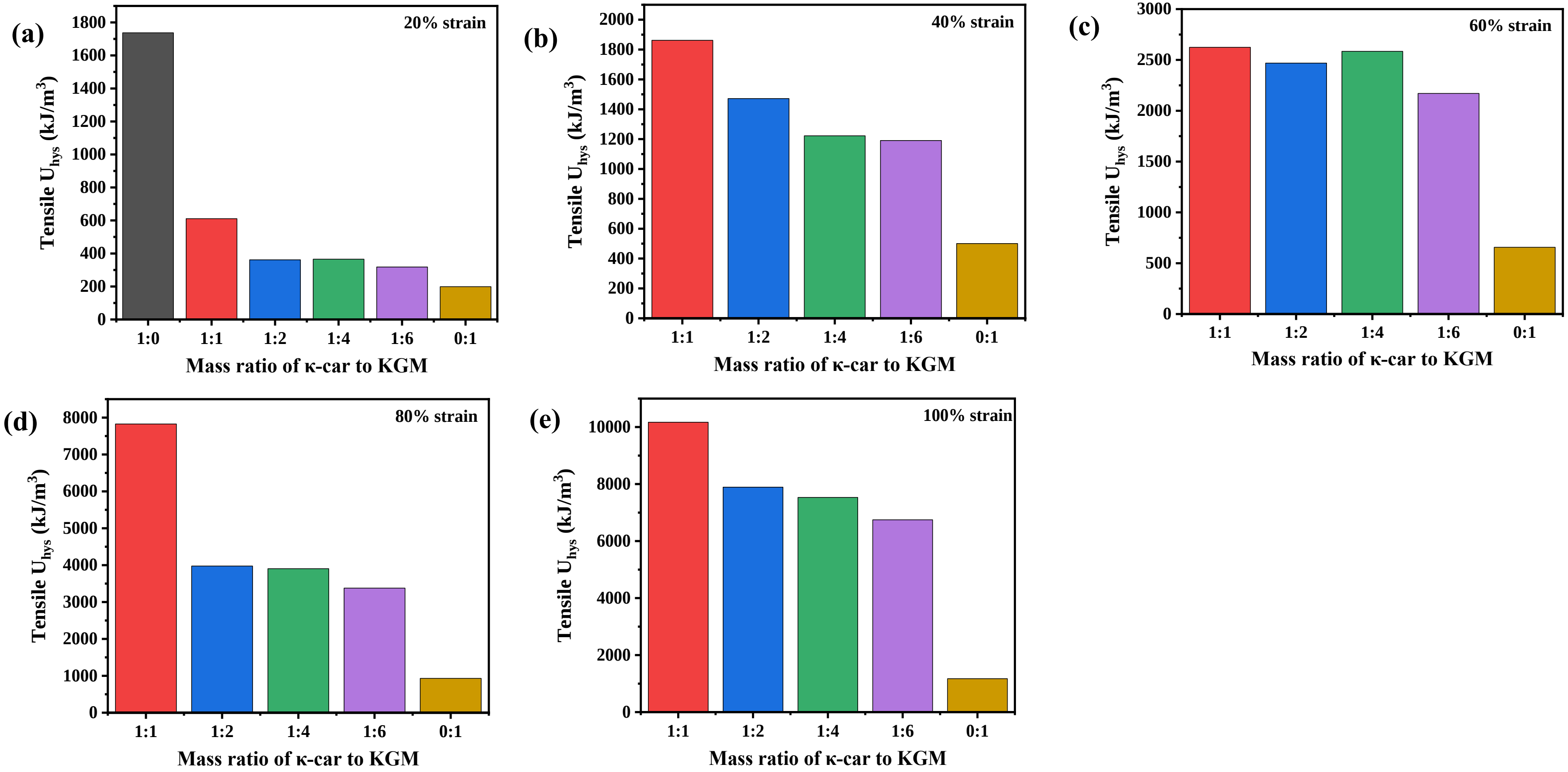

Fig. S3. The tensile U_{hys} of the κ-car-k^+/KGM gels with different mass ratio at (a) 20%, (b) 40%, (c) 60%, (d) 80%, (e) 100% strain.

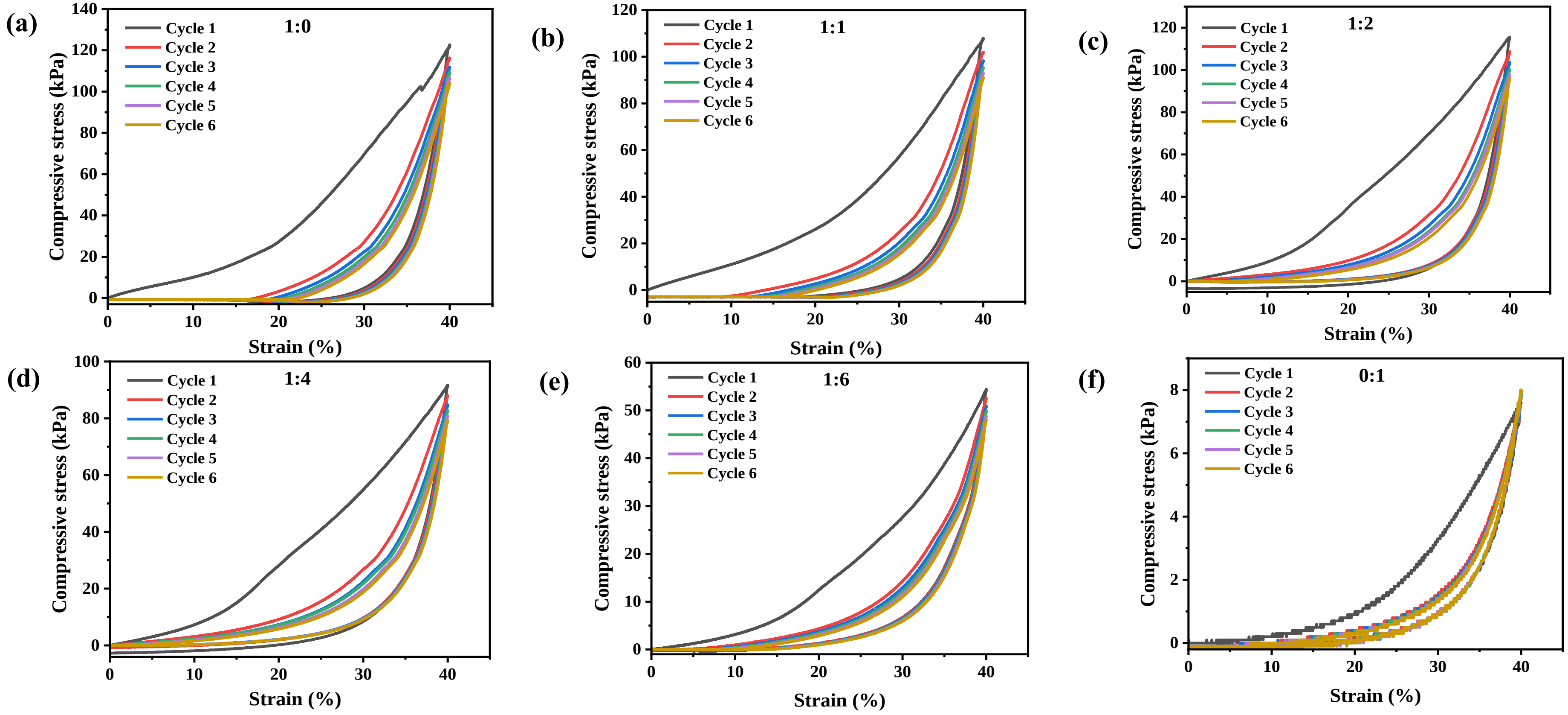

Fig. S4. The cyclic compressive loading-unloading curves of (a) κ-car-k^+ SNs, (b-e) κ-car-k^+/KGM DNs with mass ratio of 1:1 to 1:6, (f) KGM SNs with 6 times loading number at 40% strain.

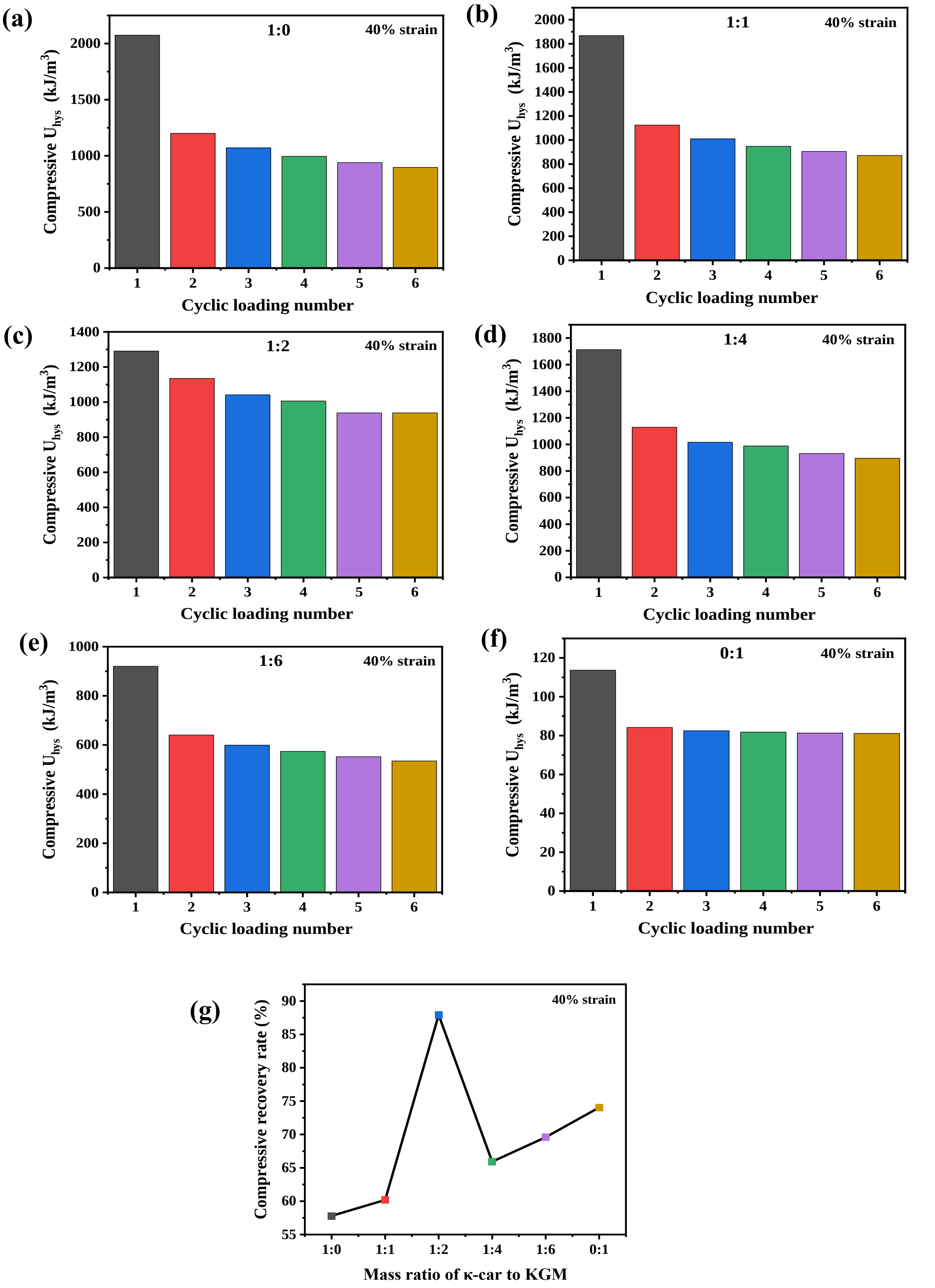

Fig. S5. The compressive U_{hys} of (a) κ-car-k^+ SNs, (b-e) κ-car-k^+/KGM DNs with mass ratio of 1:1 to 1:6, (f) KGM SNs with 6 times loading number at 40% strain, (g) compressive recovery rate of κ-car-k^+/KGM gels at 40% strain.

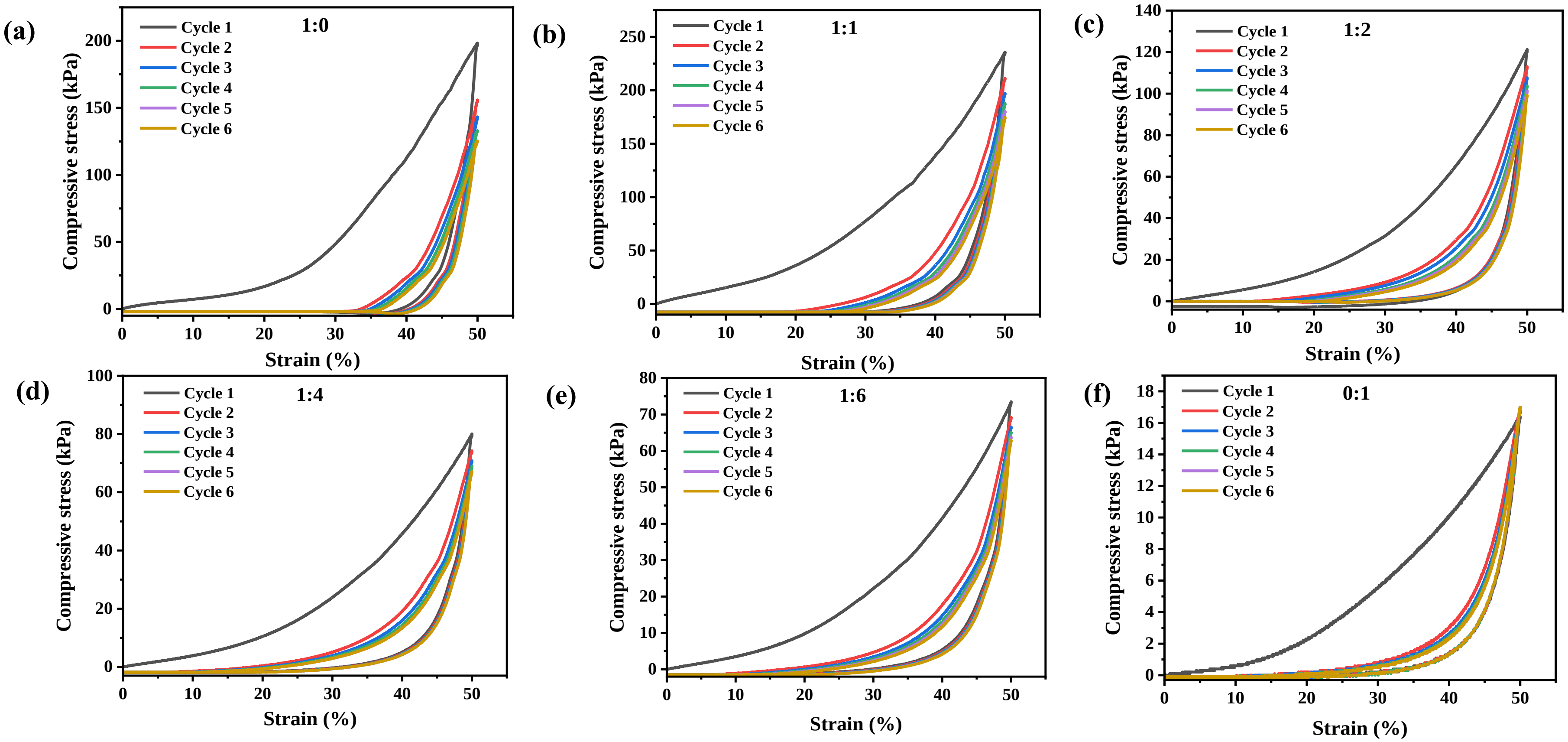

Fig. S6. The cyclic compressive loading-unloading curves of (a) κ-car-k^+ SNs, (b-e) κ-car-k^+/KGM DNs with mass ratio of 1:1 to 1:6, (f) KGM SNs with 6 times loading number at 50% strain.

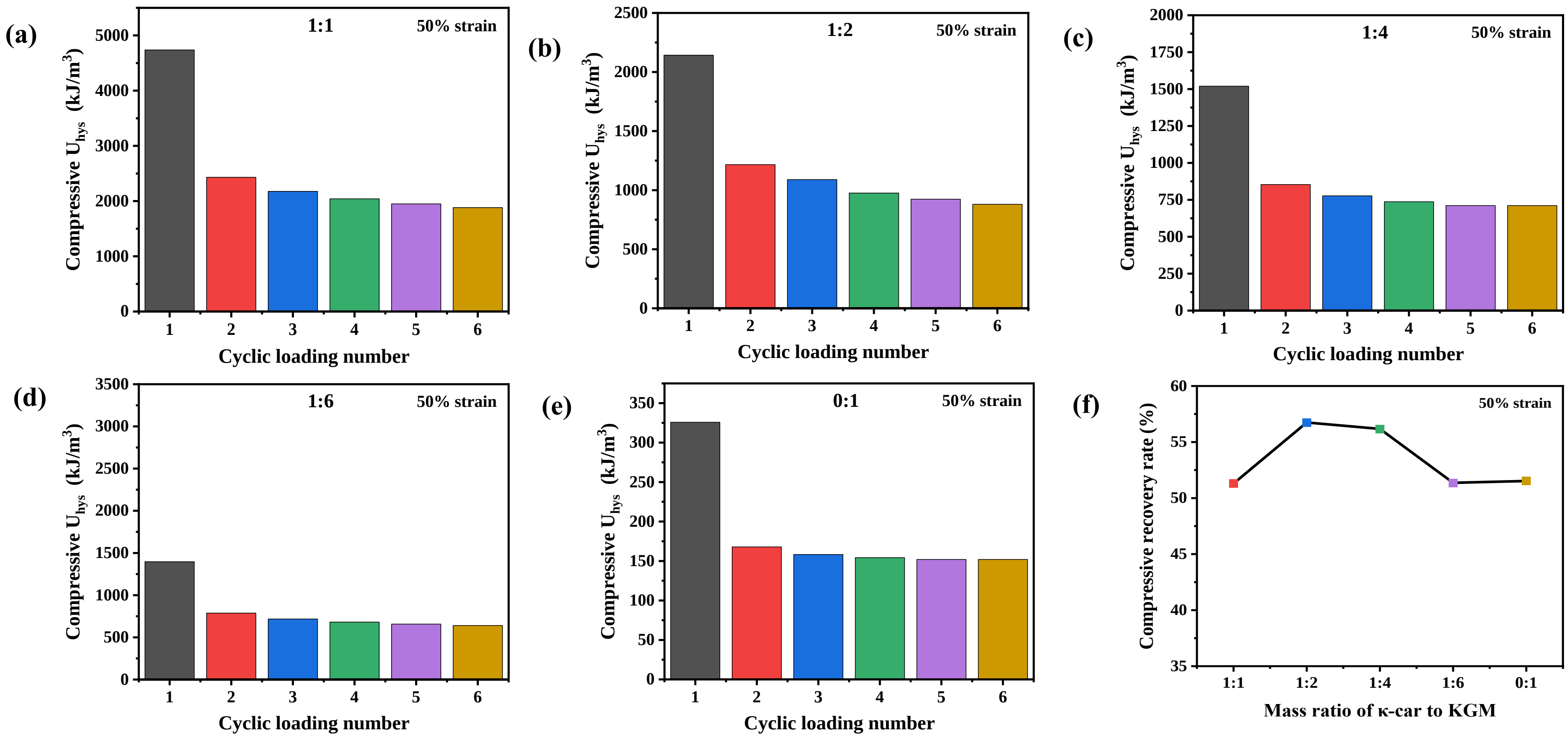

Fig. S7. The compressive U_{hys} of (a-d) κ-car-k$^+$/KGM DNs with mass ratio of 1:1 to 1:6, (e) KGM SNs with 6 times loading number at 50% strain, (f) compressive recovery rate of κ-car-k$^+$/KGM gels at 50% strain.

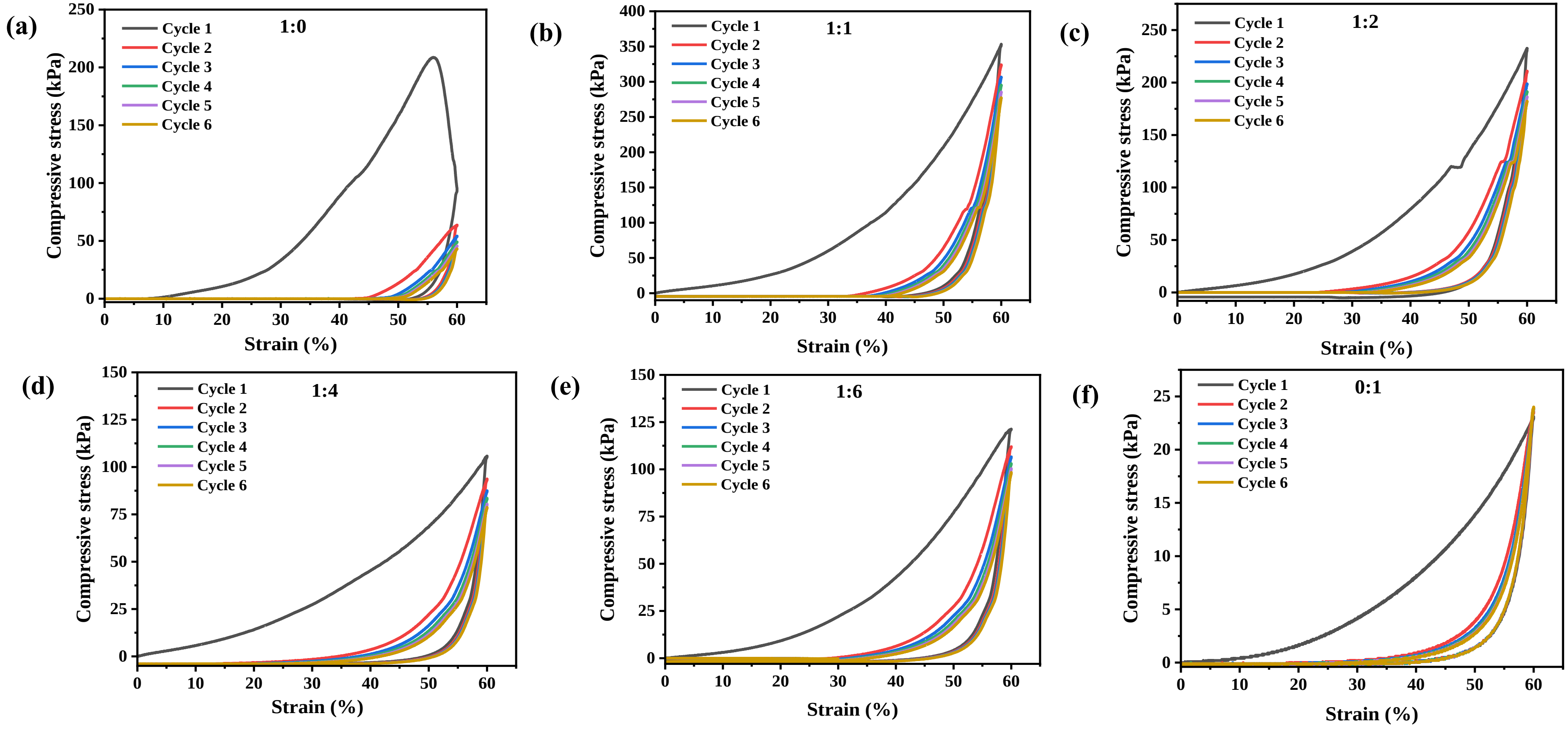

Fig. S8. The cyclic compressive loading-unloading curves of (a) κ-car-k^+ SNs, (b-e) κ-car-k^+/KGM DNs with mass ratio of 1:1 to 1:6, (f) KGM SNs with 6 times loading number at 60% strain.

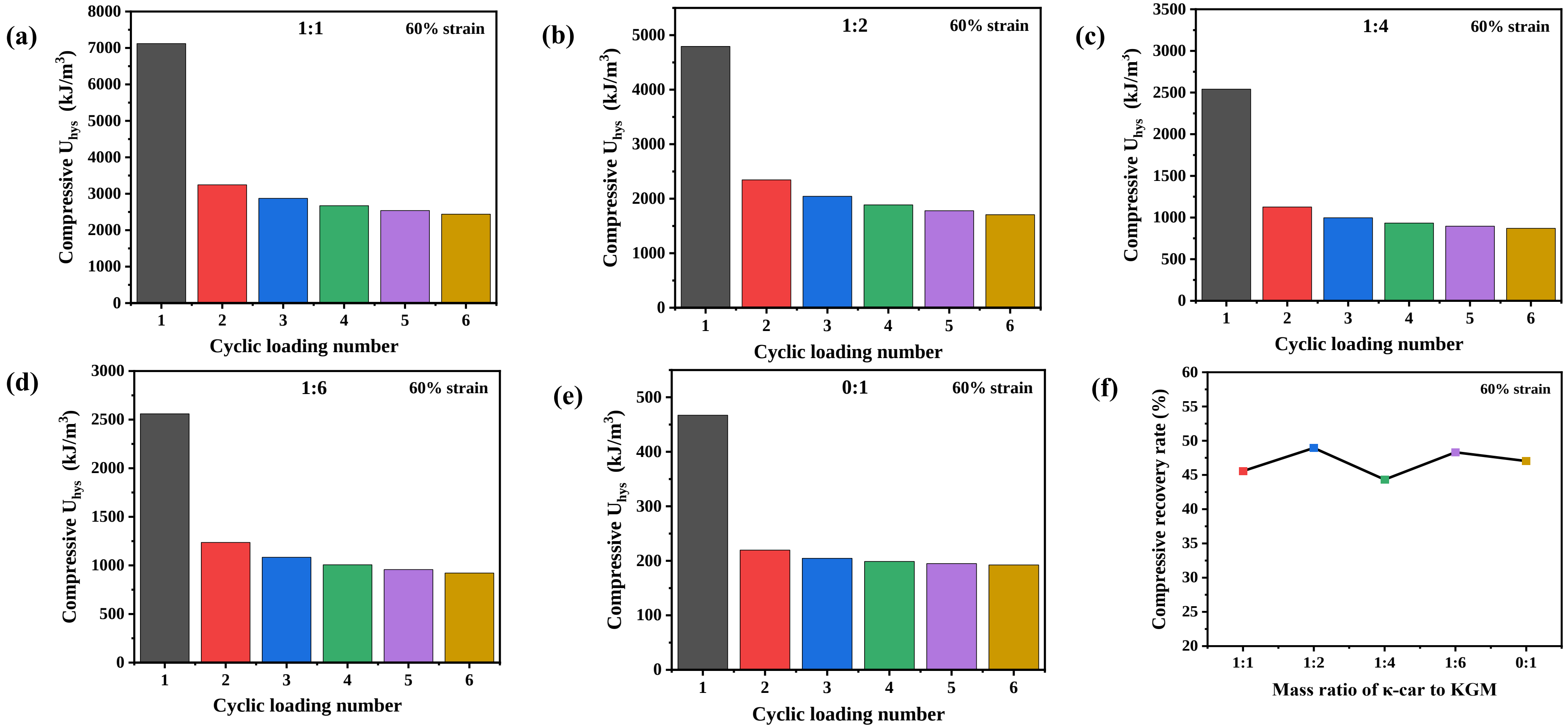

Fig. S9. The compressive U_{hys} of (a-d) κ-car-k$^+$/KGM DNs with mass ratio of 1:1 to 1:6, (e) KGM SNs with 6 times loading number at 60% strain, (f) compressive recovery rate of κ-car-k$^+$/KGM gels at 60% strain.

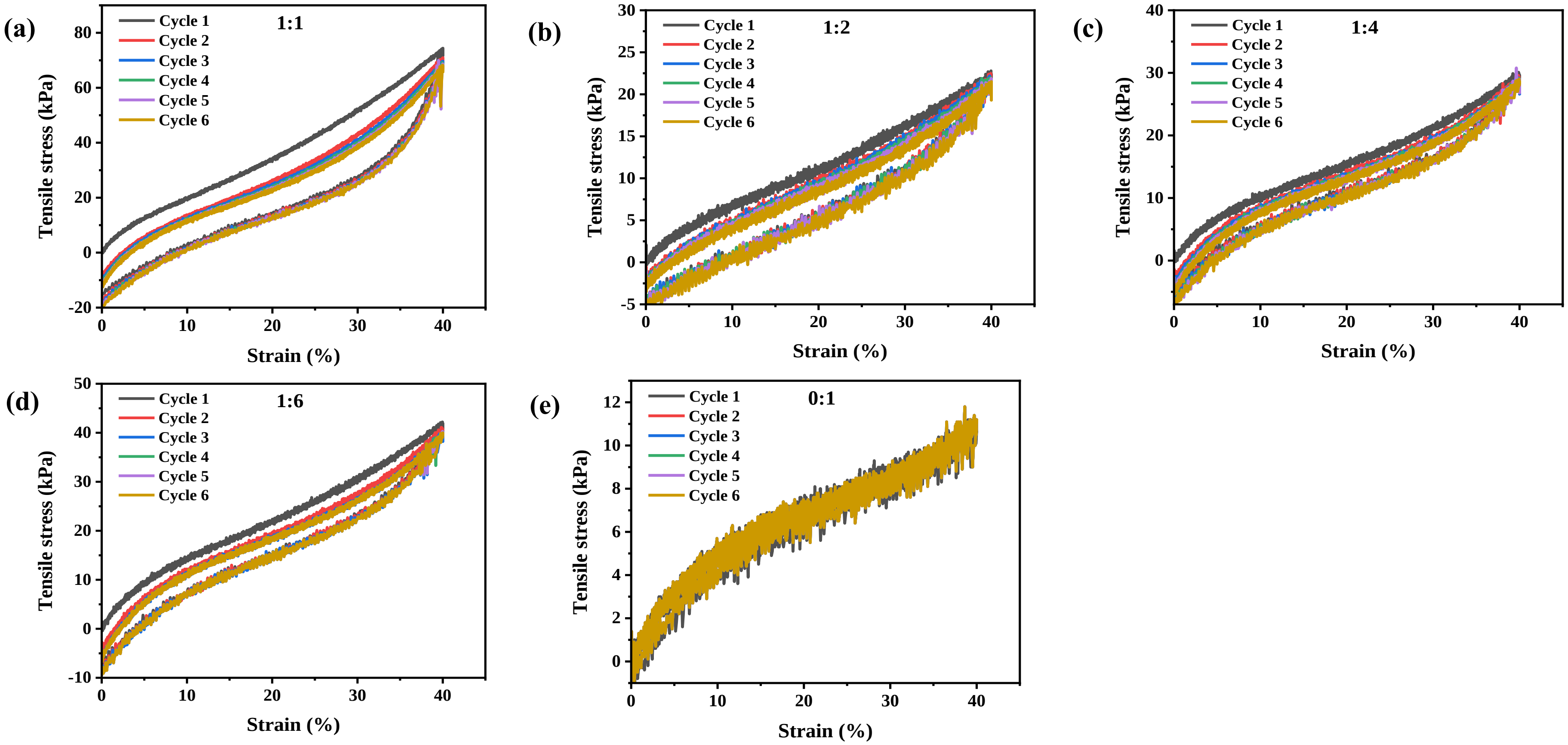

Fig. S10. The cyclic tensile loading-unloading curves of (a-d) κ-car-k$^+$/KGM DNs with mass ratio of 1:1 to 1:6, (e) KGM SNs with 6 times loading number at 40% strain.

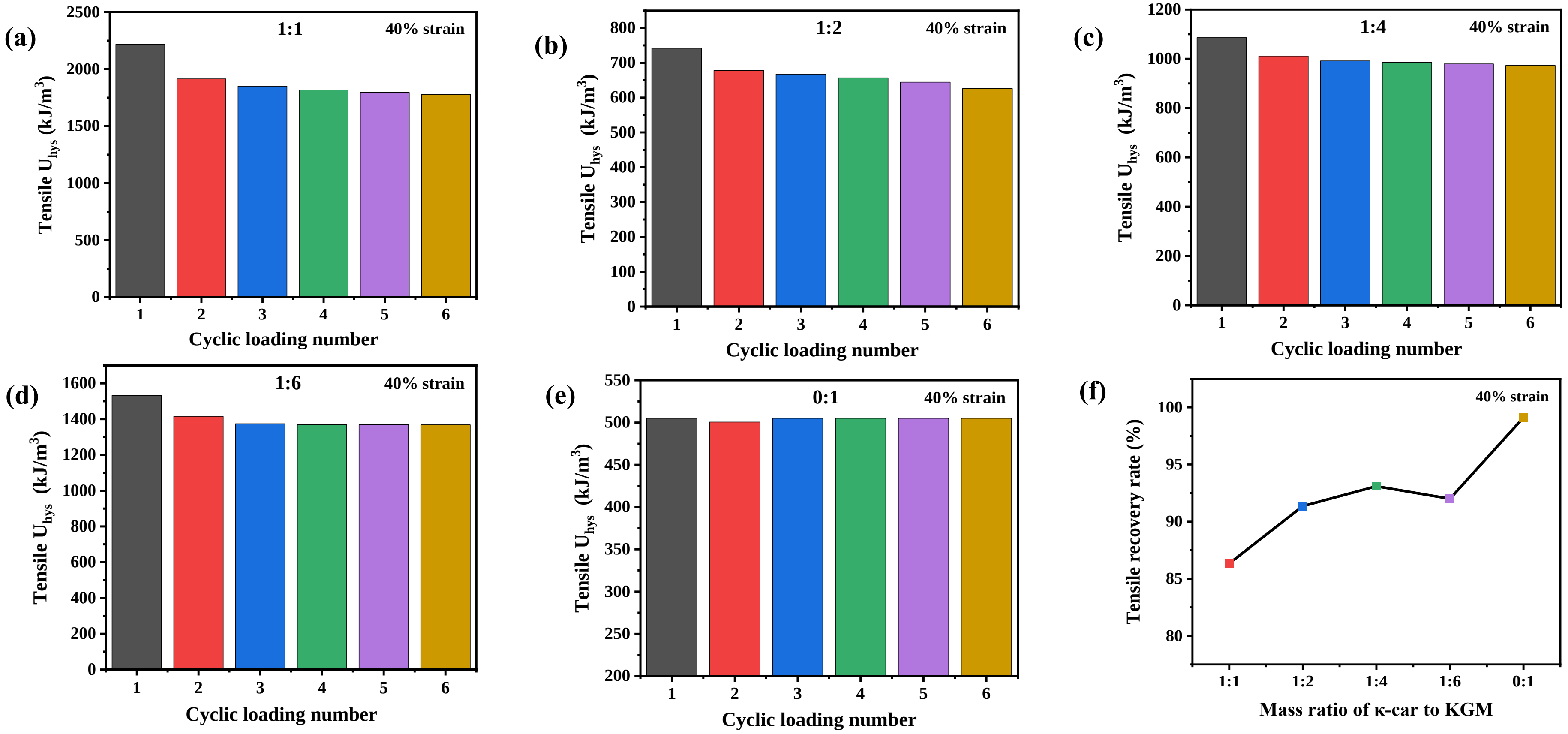

Fig. S11. The tensile U_{hys} of (a-d) κ-car-k^{+}/KGM DNs with mass ratio of 1:1 to 1:6, (e) KGM SNs with 6 times loading number at 40% strain, (f) tensile recovery rate of κ-car-k^{+}/KGM gels at 40% strain.

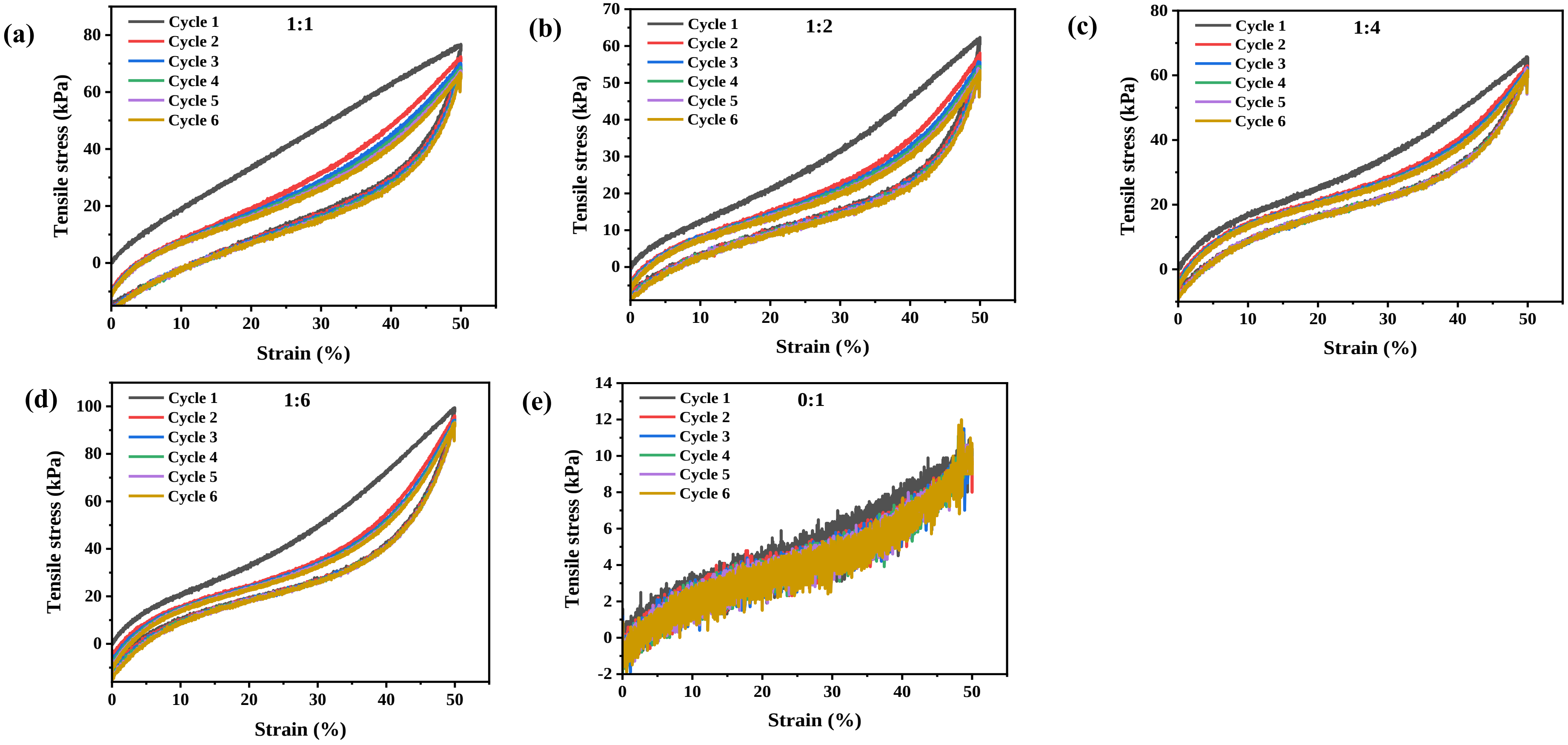

Fig. S12. The cyclic tensile loading-unloading curves of (a-d) κ-car-k^{+}/KGM DNs with mass ratio of 1:1 to 1:6, (e) KGM SNs with 6 times loading number at 50% strain.

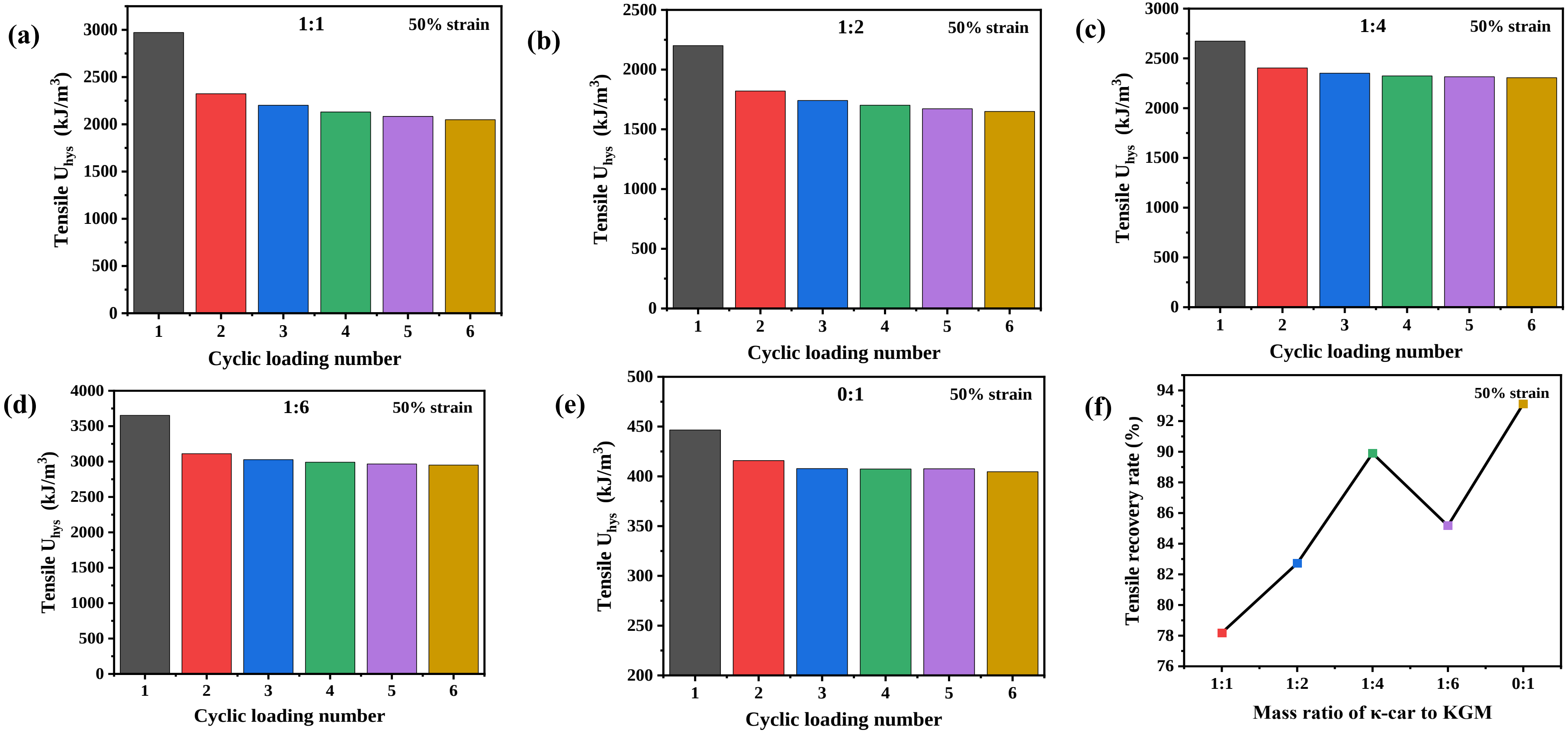

Fig. S13. The tensile U_{hys} of (a-d) κ-car-k^+/KGM DNs with mass ratio of 1:1 to 1:6, (e) KGM SNs with 6 times loading number at 50% strain, (f) tensile recovery rate of κ-car-k^+/KGM gels at 50% strain.

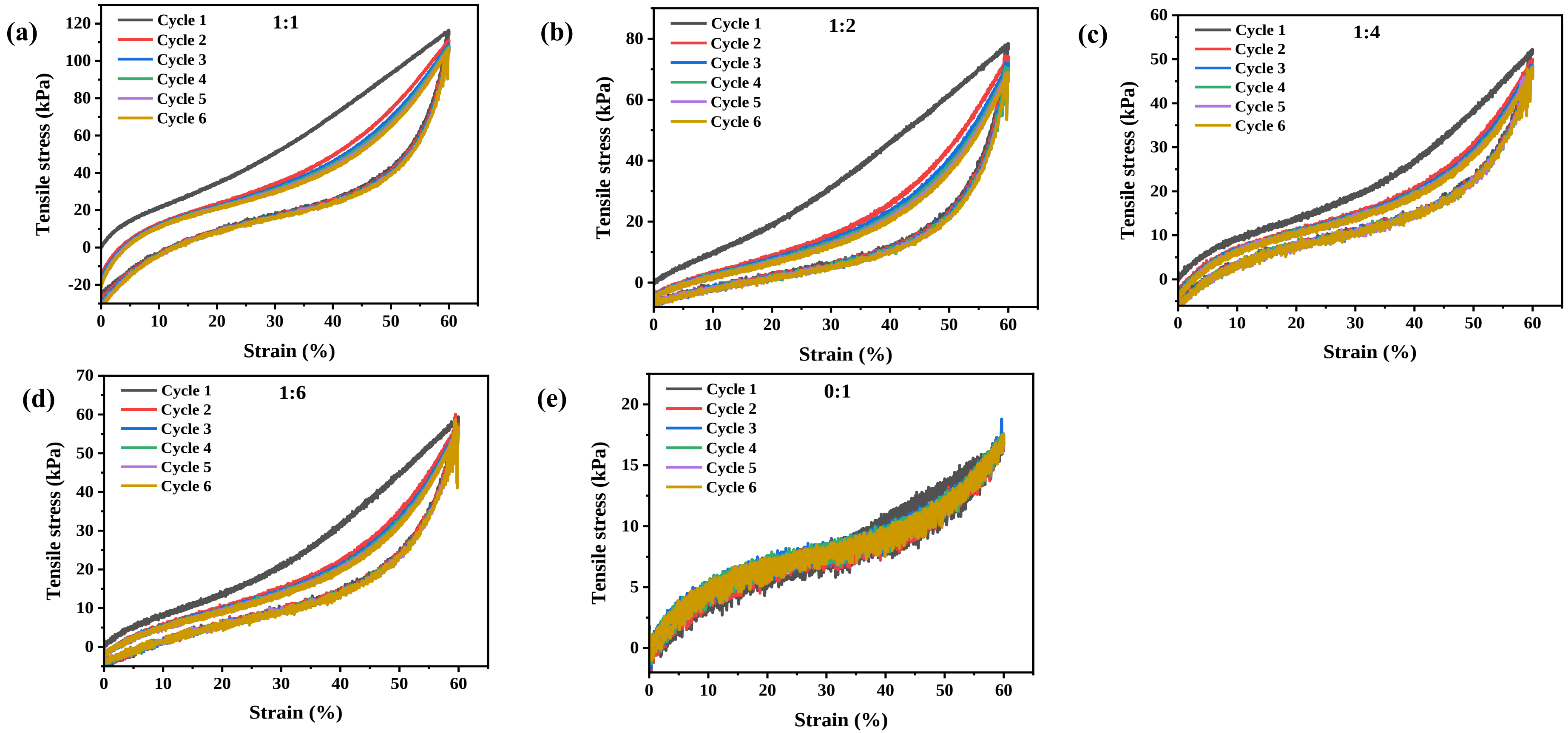

Fig. S14. The cyclic tensile loading-unloading curves of (a-d) κ-car-k$^+$/KGM DNs with mass ratio of 1:1 to 1:6, (e) KGM SNs with 6 times loading number at 60% strain.

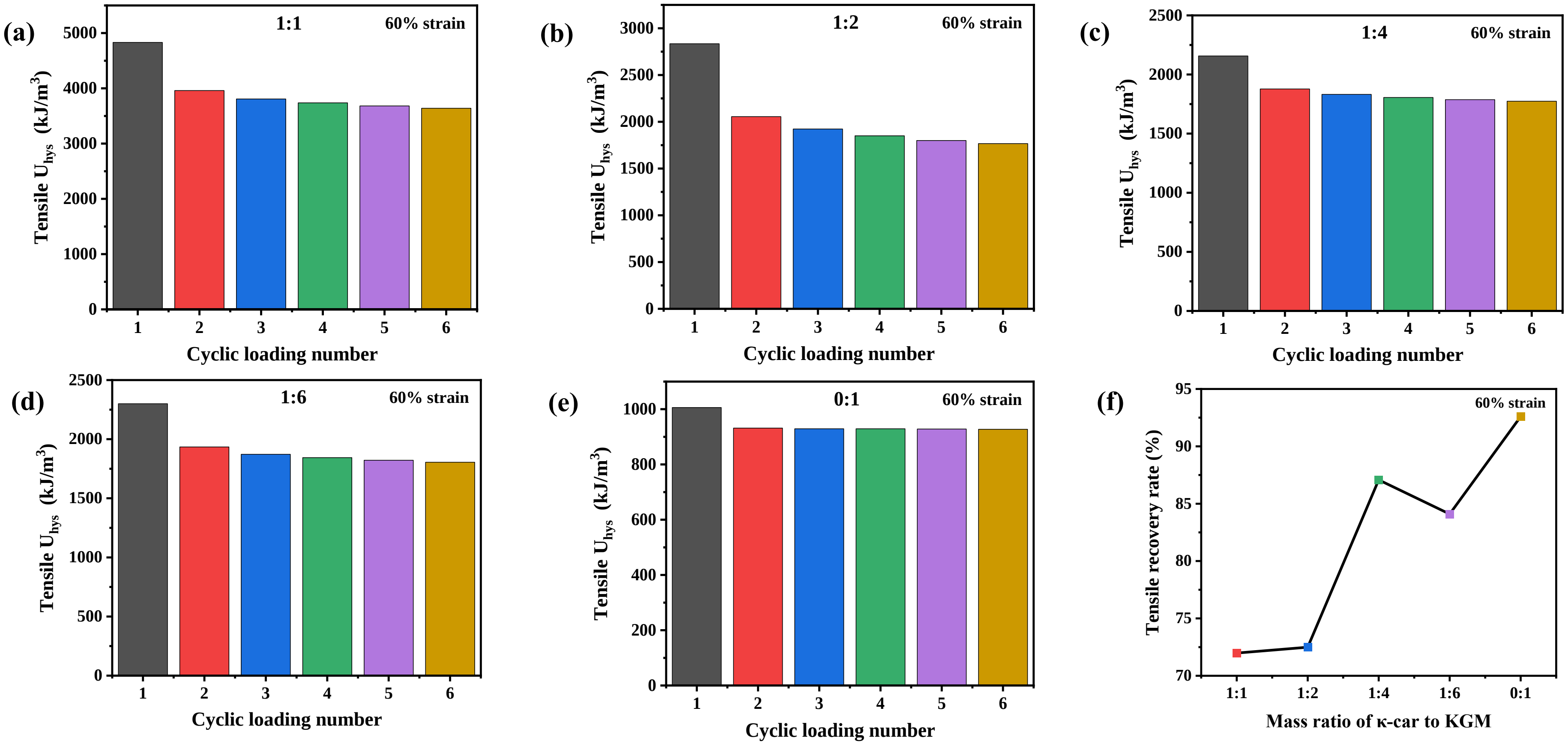

Fig. S15. The tensile U_{hys} of (a-d) κ-car-k$^+$/KGM DNs with mass ratio of 1:1 to 1:6, (e) KGM SNs with 6 times loading number at 60% strain, (f) tensile recovery rate of κ-car-k$^+$/KGM gels at 60% strain.
